# Canonical Wnt/β-Catenin Signalling Is Essential for Optic Cup Formation

**DOI:** 10.1371/journal.pone.0081158

**Published:** 2013-12-04

**Authors:** Anna-Carin Hägglund, Anna Berghard, Leif Carlsson

**Affiliations:** 1 Umeå Center for Molecular Medicine, Umeå University, Umeå, Sweden; 2 Department of Molecular Biology, Umeå University, Umeå, Sweden; National Eye Institute, United States of America

## Abstract

A multitude of signalling pathways are involved in the process of forming an eye. Here we demonstrate that β-catenin is essential for eye development as inactivation of *β-catenin* prior to cellular specification in the optic vesicle caused anophthalmia in mice. By achieving this early and tissue-specific *β-catenin* inactivation we find that retinal pigment epithelium (RPE) commitment was blocked and eye development was arrested prior to optic cup formation due to a loss of canonical Wnt signalling in the dorsal optic vesicle. Thus, these results show that Wnt/β-catenin signalling is required earlier and play a more central role in eye development than previous studies have indicated. In our genetic model system a few RPE cells could escape *β-catenin* inactivation leading to the formation of a small optic rudiment. The optic rudiment contained several neural retinal cell classes surrounded by an RPE. Unlike the RPE cells, the neural retinal cells could be β-catenin-negative revealing that differentiation of the neural retinal cell classes is β-catenin-independent. Moreover, although dorsoventral patterning is initiated in the mutant optic vesicle, the neural retinal cells in the optic rudiment displayed almost exclusively ventral identity. Thus, β-catenin is required for optic cup formation, commitment to RPE cells and maintenance of dorsal identity of the retina.

## Introduction

The vertebrate eye develops through a series of co-ordinated interactions between tissues of different embryonic origin. The eye field is specified in the anterior neural plate immediately following gastrulation [1]. The lateral walls of the diencephalon then evaginate resulting in the optic vesicle [2]. The distal portion of the optic vesicle makes contact with the surface ectoderm which initiates the formation of the lens placode. Reciprocal interactions between the lens placode and the optic vesicle promote the formation of the optic cup [3]. However, such inductive interactions might not be strictly necessary since it has been shown recently that the optic vesicle can form the optic cup by a self-organising mechanism that is independent of external cues from the lens placode [4]. Lens morphogenesis, establishment of dorsoventral polarity and specification of the neural retina, retinal pigment epithelium (RPE) and optic stalk occurs concurrently with the transformation of the optic vesicle to optic cup [3].

Specification of different cell types in the eye is mediated by a number of key paracrine signalling molecules. Early neural retina specification is mediated by fibroblast growth factor (FGF) emanating from the surface ectoderm in the prospective lens placode leading to expression of the transcription factor Vsx2 (also Chx10) in the lateral part of the optic vesicle [5]. There is evidence to suggest that RPE cells are specified by the transforming growth factor β (TGFβ) family member Activin A which is secreted by the extraocular mesenchyme [6]. The RPE cell is a versatile cell type and is involved in many important aspects of eye physiology [7], although whether RPE cells, once specified, influence the development of other cell types within the eye is unclear. The RPE cells are specified in the optic vesicle before pigmentation. The *Microphthalmia-associated transcription factor* (*Mitf*) is essential for RPE cell development but is expressed throughout the early optic vesicle where it marks undifferentiated bipotential neuroepithelial precursor cells and following cellular commitment becomes restricted to RPE cells [8]. The *Orthodenticle homolog 2* (*Otx2*) transcription factor is expressed in the eye field before RPE specification but later becomes restricted to the RPE cells [9], [10]. *Otx2* is required for *Mitf* expression and activates genes important for pigmentation in co-operation with Mitf [8], [10]. In contrast to Mitf, Otx2 is also important for formation of specific cell populations in the neural retina such as photoreceptor cells [11].

During the transformation of the optic vesicle into the optic cup, the opposing actions of bone morphogenetic protein (Bmp) and hedgehog signalling are thought to generate dorsoventral patterning. Hedgehog signalling has been implicated in the specification of ventral structures such as the optic stalk [12], whereas Bmp signalling has also been shown to be involved in optic vesicle development and lens placode induction [13], [14], [15], [16]. The *Bmp4* gene is expressed in the dorsal part of the optic vesicle and is involved in dorsal patterning [15], [17]. The establishment of dorsoventral identity in the neural retina is manifested by the transcription factors *Tbx5* and *Vax2*, expressed dorsally and ventrally, respectively [18]. The dorsoventral patterning of the neural retina is critical for correct topographic projection of retinal ganglion cell axons to the optic tectum in the brain, and for establishing a cone opsin gradient in the neural retina [19], [20].

In addition to dorsoventral patterning the neural retina is composed of functionally distinct cell types organised into a laminar structure. The different cellular layers are referred to as follows (from outside-in), the outer nuclear layer, the inner nuclear layer and the ganglion cell layer. Photoreceptor proteins (opsins) are activated by light (photons) in the rods and cones that are in the outer nuclear layer. The inner nuclear layer consists of amacrine cells, bipolar cells and horizontal cells that essentially transfer and modulate information from the outer nuclear layer to the ganglion cell layer. The ganglion cell layer consists of retinal ganglion cells which are projection neurons and convey visual input information from the retina along the optic nerve to the brain [21]. The different cell classes in the neural retina are formed from one progenitor cell type during embryonic development, in a specific developmental order that is evolutionary conserved. For example, the retinal ganglion cells are generated first, and rods and bipolar cells are generated last [22], [23], [24], [25], [26].

β-catenin has been shown to be involved in different aspects of eye development. The β-catenin molecule has two different cellular functions; to regulate cellular adhesion by interacting with cadherins, and to mediate the canonical Wnt signalling pathway [27]. In the absence of a Wnt ligand and if not bound to cadherins, β-catenin is phosphorylated and actively degraded by a multiprotein destruction complex [28]. Binding of Wnt ligands to Frizzled (Fz) transmembrane receptors and Lrp5/6 co-receptors starts a series of events leading to inhibition of phosphorylation of β-catenin [29]. The unphosphorylated β-catenin accumulates in the cytoplasm and can become translocated to the nucleus where is acts as a transcriptional activator together with DNA-binding TCF/Lef transcription factors [30]. Canonical Wnt signalling conveyed by β-catenin in the RPE cells is important for maintenance of dorsal identity in zebrafish retina [31], and inactivation of *Lrp6*, a co-receptor of canonical Wnt signalling, causes a defect in dorsal retinal patterning leading to a coloboma [32], [33]. In studies where β-catenin function was investigated in mice it was inactivated in the already specified RPE layer in the optic cup, revealing that it was important for the maintenance of these cells [34], [35]. Moreover, the function of β-catenin in cell adhesion is essential for the lamination of the neural retina [36]. However, the role of β-catenin in early patterning of the optic vesicle/cup has not been directly investigated. To address this issue we have used a novel genetic model to inactivate *β-catenin* in early eye progenitor cells prior to cellular commitment in the optic vesicle. This approach revealed that β-catenin is essential for eye development since the optic vesicle is unable to transform into the optic cup. This phenotype is most likely due to that the RPE layer is not specified at the correct time during eye development.

## Materials and Methods

### Ethics Statement

The mice were maintained at the animal facility at Umeå University and all experiments involving animals were approved by the Animal Review Board at the Court of Appeal of Northern Norrland in Umeå.

### Generation and Maintenance of Mice

The *Lhx2-Cre* transgenic mouse, *β-catenin^flox/flox^* mice (exons 2–6 were flanked by loxp sites) and *ROSA26R* mice has been described previously [37] [38] [39]. The *β-catenin* germ-line null allele was generated by crossing *β-catenin^flox/+^* with *Zp3-Cre* transgenic mice which inactivate genes in the female germ-line [40]. The *Lhx2-Cre:β-catenin^flox/flox^* and the *Lhx2-Cre:β-catenin^flox/^* ^−^ mice are born but die prior to postnatal day 2 (P2) of unknown causes. The genotypes of the control mice in these crosses were; *Lhx2-Cre:β-catenin^+/^* ^−^, *Lhx2-Cre:β-catenin^flox/+^*, *β-catenin^flox/flox^*, *β-catenin^flox/^* ^−^, *β-catenin^flox/+^* and *β-catenin^+/^* ^−^. Genotyping was performed by PCR analysis of genomic DNA extracted from tail biopsies using the following primer pairs: *Lhx2-Cre* transgene: Lhx2CreF: 5′-TTCCACAGTCTGTCGGGC-3′ and Lhx2CreR: 5′-CCTGGCGATCCCTGAACATGT-3′. *β-catenin^flox^* and the *β-catenin* ^−^ alleles, IMR1512: 5′-AAGGTAGAGTGATGAAAGTTGTT-3′, IMR1513: 5′-CACCATGTCCTCTGTCTATTC-3′ and RM43: 5′-TACACTATTGAATCACAGGGACTT- 3′. *ROSA26R* mice, IMR8052: 5′-GCGAAGAGTTTGTCCTCAACC-3′, IMR8545: 5′-AAAGTCGCTCTGAGTTGTTAT-3′ and IMR8546: 5′-GGAGCGGGAGAAATGGATATG-3′. The morning of the vaginal plug was considered as embryonic day 0.5 (E0.5).

### Histology, *in situ* Hybridisation and Immunohistochemistry

Embryos were isolated and fixed in 4% (w/v) paraformaldehyde (PFA) in PBS at 4°C. Embryos used for *in situ* hybridisation were fixed between 30 minutes to 2 hours. After fixation the embryos were transferred to 30% (w/v) sucrose in PBS for 24 hours at 4°C, mounted in Tissue-tek (Sakura) and stored at −80°C. Sectioning (8–10 µm) was performed on a cryostat (Microm HM505E) and collected on superfrost plus slides (Menzel-Gläser). *In situ* hybridisation using DIG labelled probes were performed essentially as previously described [41]. The following probes were used: β-catenin (BC048153, nucleotides 170–758 that covers exons 2–5 which are deleted when *β-catenin* is inactivated), Lhx2 (NM_010710, nucleotides 460–1750), BMP4 (NM_007554, nucleotides 117–578), Pax6 (NM_013627, nucleotides 799–1605), Pax2 (IMAGE clone: 40142573), Rx (IMAGE clone: 5366450), Sox2 (IMAGE clone: 6413283), Otx2 (NM_144841, nucleotides 338–1158), Six6 (NM_011384, nucleotides 126–932), Six3 (BC098096, nucleotides 771–1222), Vsx2 (IMAGE clone: 6492679), Mitf (IMAGE clone: 40047440), Axin2 (BC057338, nucleotides: 1–1520), Tbx5 (BC090639, nucleotides 1–2924), Raldh1 (BC054386, nucleotides 755–2078), Vax2 (IMAGE clone: 40101825), Nr2f1 (IMAGE clone: 6511382), Nr2f2 (IMAGE clone: 6829692), Nrl (BC031440, nucleotides 1–1123), Crx (BC016502, nucleotides 1–1728), Trβ2 (IMAGE clone: 40057540) and Wnt2b (IMAGE clone: 8734027).

For hematoxylin-eosin staining, tissue sections were incubated sequentially in the following solutions; Mayer’s hematoxylin solution for 2 minutes, water for 15 minutes, eosin solution for 2 minutes, 95% ethanol for 2×1 minutes, in 99% ethanol for 2×1 minutes and in xylene for 5 minutes. The slides were mounted with DPX mounting media (VWR). To get an approximate value of the relative eye size for the different genotypes, the largest diameter of the control eyes and the mutant optic rudiment at different ages were measured and compared.

Immunohistochemistry was performed essentially as previously described [42]. Embryos fixed in 4% PFA for 30–60 minutes were cryosectioned 8–10 µm. Slides were washed 3×5 minutes in TBS (50 mM Tris-HCl pH 7.4, 150 mM NaCl) and blocked with 10% FCS in TBST (TBS with 0,1% Triton X-100) for 20 minutes. The primary antibody, diluted in TBST containing 5% (v/v) FCS, was applied to slides and incubated over night at 4°C. The slides were subsequently washed in TBST (3×5 minutes) and the secondary antibody containing DAPI was applied and incubated at room temperature for 1 hour. Slides were washed in TBST (3×5 minutes) and mounted with fluorescence mounting medium (Vectashield, Vector Laboratories). The primary antibodies used were; goat anti-Brn3 (sc-6026, Santa Cruz Biotechnology Inc; dilution 1∶50), rabbit anti-β-galactosidase (55976, ICN Pharmaceuticals; dilution; 1∶1000), rabbit anti-β-catenin (C2206, Sigma-Aldrich; dilution1∶2000), rabbit anti-Prox1 (11-002P, AngioBio; dilution 1∶1000), rabbit anti-Ptf1a (a gift from Helena Edlund; dilution 1∶800), mouse anti-Mitf (X1405M, Exalpha Biologicals; dilution 1∶400), rabbit anti-pMLC2 (3671, Cell Signalling; dilution 1∶500, phospho-Myosin light chain 2, Ser19), rabbit anti-activated Caspase 3 (ab13847, Abcam; dilution 1∶1000) and rat anti-N-cadherin (MNCD2, DSHB; dilution 1∶100). To detect N-cadherin, sections were immersed in HEPES-buffered saline and heated in a microwave oven for three minutes before blocking [43]. F-actin was detected using FITC-labeled phalloidin (P2141, Sigma-Aldrich; dilution 1∶100). The following secondary antibodies were used for immunofluorescence: Cy3-conjugated donkey anti-rabbit (711-165-152, Jackson ImmunoResearch Laboratories Inc.; dilution 1∶1000), Alexa Fluor 488-conjugated donkey anti-rat (A21208, Molecular Probes; dilution 1∶1000), Alexa Fluor 488-conjugated goat anti-mouse (A21121, Molecular Probes; dilution 1∶1000) and Alexa Fluor 488-conjugated donkey anti-goat (A11055, Molecular Probes; dilution 1∶1000). Bright field or fluorescence optical images were captured using a Nikon Eclipse E800 microscope with a Nikon DS-Ri1 digital colour camera. Confocal images were taken with a Zeiss LSM 710 microscope.

### BrdU Labelling and Quantification of BrdU^+^ and Activated Casp-3^+^ Cells

Pregnant mice were injected intraperitoneally with 5-bromo-2′-deoxyuridine (BrdU; Sigma) at 50 µg/g body weight 1 hour before sacrifice and collection of embryos. Embryos were fixed for 1 hour in 4% (w/v) PFA. After sectioning, the slides were washed in PBS and then denatured in 2 M HCl for 30 minutes at 37°C. The slides were subsequently neutralised with 0,1 M Borate buffer (pH 8,5), followed by a standard immunostaining as described above using mouse anti-BrdU (560210, BD Pharmingen; dilution 1∶20). The relative number of BrdU^+^ cells (e.g. BrdU^+^ cell/DAPI^+^ cell) was counted in comparable areas in the prospective neural retina in control and *Lhx2-Cre:β-catenin^flox/flox^* embryos. The absolute numbers of activated Casp-3^+^ cells was counted in prospective neural retina in control and *Lhx2-Cre:β-catenin^flox/flox^* embryos. The data presented are based on two sections from both optic vesicles in three control and three mutant embryos. Data are presented as mean ± standard deviation (SD). p values were calculated using Student’s t test.

## Results

### Mouse Embryos with *β-catenin* Inactivated in the Early Optic Vesicle are Anophthalmic

Previous studies have shown that β-catenin is important for maintaining RPE cell identity in the mouse and to indirectly maintain dorsal identity of the zebrafish retina [31], [34], [35]. To address the functional role of β-catenin in mammals during early patterning of the optic vesicle we inactivated β-catenin prior to cellular commitment in the optic vesicle. To inactivate *β-catenin* in the early optic vesicle we crossed mouse lines harbouring a null allele or floxed alleles of the *β-catenin* gene [38] (*β-catenin^+/^* ^−^ or *β-catenin^flox/flox^* mice) to *Lhx2-Cre* transgenic mice. In the *Lhx2-Cre* transgenic mouse embryos Cre recombinase is expressed within the first progenitor cells committed to eye development in the anterior neural plate [37]. This approach therefore enables evaluation of primary and direct effects due to *β-catenin* inactivation in the early eye and not secondary effects resulting from defects in forebrain development [37]. The mice with the genotypes *Lhx2-Cre:β-catenin^flox/flox^* and *Lhx2-Cre:β-catenin^flox/^* ^−^ will both result in inactivation of *β-catenin* in the early eye-committed progenitor cells, but since the severity of their respective phenotypes differ they will be referred to by their full genotype. The phenotype observed in embryos of other possible genotypes generated in these crosses was indistinguishable from wild type and will be referred to as “control mice” (see Material and Methods).

**Figure 1:**
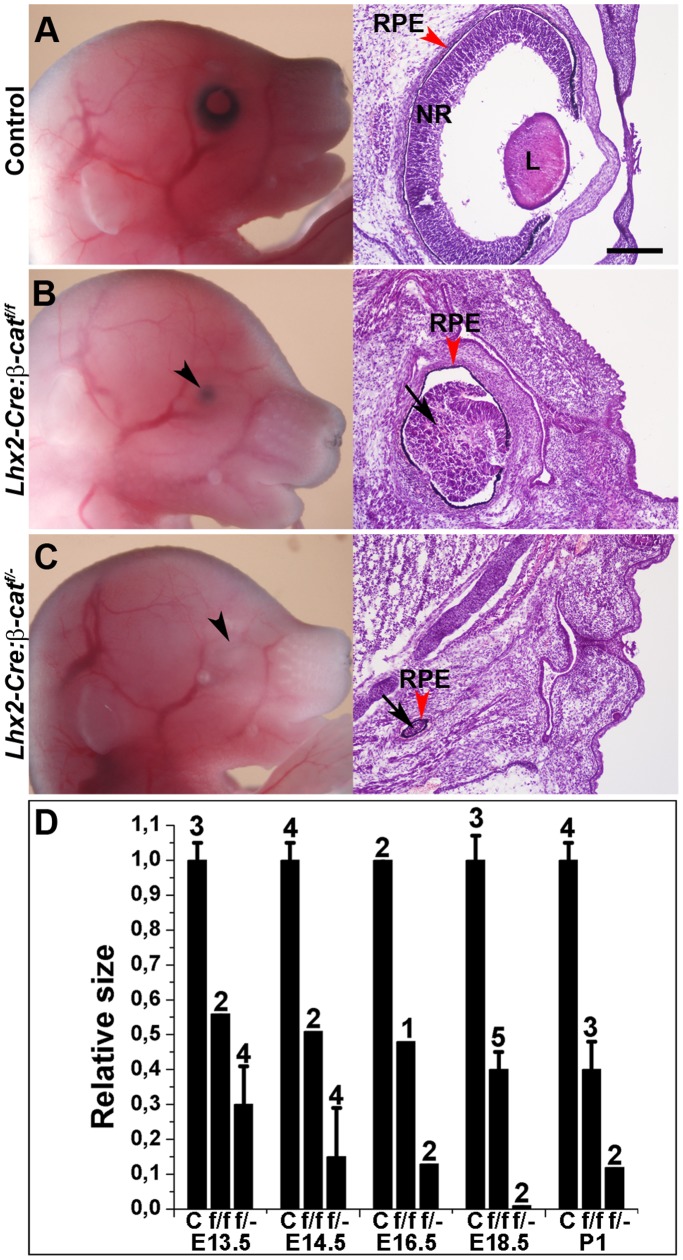
β-catenin mutant embryos are anophthalmic. (A–C) The left panels show lateral views of E16.5 embryos of control (A), *Lhx2-Cre:β-catenin^flox/flox^* (β-cat^f/f^) (B) and *Lhx2-Cre:β-catenin^flox/^* ^−^ (*β-cat^f/^* ^−^) (C) embryos. Arrow heads indicate where the eye should be located in the mutant embryos. The right panels show hematoxylin/eosin staining of coronal tissue sections of the same embryos. Black arrows indicate the optic rudiment surrounded by RPE cells (red arrow heads) that can develop in the mutant embryos. NR: neural retina. L: lens. RPE: retinal pigment epithelium. (D) Relative size of the optic vesicle-derived structure in the *Lhx2-Cre:β-catenin^flox/flox^* (*f/f*) embryos and *Lhx2-Cre:β-catenin^flox/^* ^−^ (*f/−*) embryos compared with control embryos (c) at the indicated embryonic age and postnatal day 1 (P1). To get an approximate value of the relative eye size for the different genotypes, the largest diameter of the control eyes and the mutant optic rudiment at different ages were measured and compared. The diameter of the eye in control embryos at each developmental stage is arbitrarily defined as 1.0. The number of eyes analysed for each genotype at the respective age is indicated for each group. SD is indicated when applicable. Scale bar: 200 µm.

Eye development was severely affected in all individuals from both the *Lhx2-Cre:β-catenin^flox/flox^* (n = 30) and *Lhx2-Cre:β-catenin^flox/^* ^−^ (n = 9) embryos (Figure 1B, 1C, left panels) whereas none of the control mice (n = 156) revealed any defect in eye development (Figure 1A), Histological analysis revealed that both the *Lhx2-Cre:β-catenin^flox/flox^* and *Lhx2-Cre:β-catenin^flox/^* ^−^ embryos were anophthalmic and lacked a lens but instead developed a small optic rudiment with no organised eye structures (Figure 1B, 1C, right panels). The most severely affected embryos lacked most optic vesicle-derived eye structures, except for a few pigmented cells (Figure 1C, right panel), and we were unable to detect any cells derived from the optic vesicle in some *Lhx2-Cre:β-catenin^flox/^* ^−^ embryos (n = 2) (data not shown). The optic rudiment was smaller in the *Lhx2-Cre:β-catenin^flox/^* ^−^ compared with the *Lhx2-Cre:β-catenin^flox/flox^* embryos at all developmental stages analysed (Figure 1D). Hence, eye development in the *Lhx2-Cre:β-catenin^flox/^* ^−^ embryos was consistently more severely affected compared with the *Lhx2-Cre:β-catenin^flox/flox^* embryos. Thus, inactivation of the *β-catenin* gene in the early optic vesicle causes anophthalmia showing that β-catenin is essential for eye development.

### The Optic Vesicle is Unable to Transform into an Optic Cup in *β-catenin* Mutant Embryos

**Figure 2:**
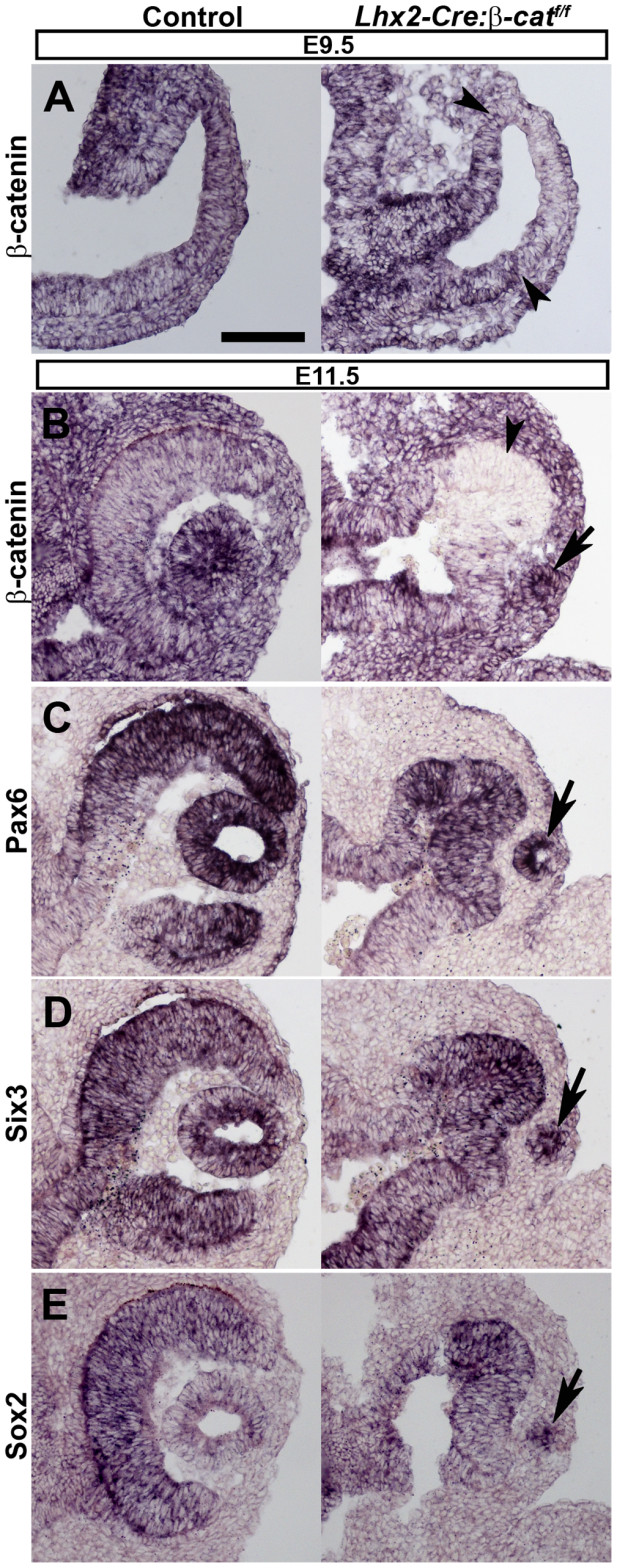
*β-catenin* mutant optic vesicles do not form an optic cup. (A) *In situ* hybridization analyses of *β-catenin* expression on coronal sections of optic vesicles of an E9.5 control embryo (left panel) and an *Lhx2-Cre:β-catenin^flox/flox^* embryo (right panel). The arrow heads indicate the boundaries of the area in the optic vesicle where *β-catenin* has been inactivated. (B–E) *In situ* hybridization analyses for gene expression of the indicated genes on coronal sections of control embryos (left panels) and *Lhx2-Cre:β-catenin^flox/flox^* embryos (right panels) at E11.5. Arrows indicate the lens pit where the early lens ectoderm remains attached to the epidermal ectoderm in mutants. Arrow head in (B) indicate the area where the *β-catenin* gene has been inactivated. Scale bar: 100 µm.

In order to identify the underlying cause of the anophthalmia we examined at which time point eye development was first perturbed. Although *β-catenin* was inactivated in the optic vesicle at E9.5 (Figure 2A), the optic vesicle in mutant and control embryos were indistinguishable at this stage (Figure 2A). However, the subsequent transformation into the optic cup did not occur in either the *Lhx2-Cre:β-catenin^flox/^* ^−^ or the *Lhx2-Cre:β-catenin^flox/flox^* mutant embryos (n = 6 and n = 14, respectively, Figure 2B–E). In the mutants the invagination of the epidermal ectoderm is initiated leading to the formation of the lens pit, but this process is delayed compared to the control (Figure 2B–E). This suggests that the lens placode is induced in the mutants and consistent with that we observe expression of lens-specific transcription factors such as *Pax6*, *Six3* and *Sox2* in this presumptive lens tissue (Figure 2C–E). Although *β-catenin* is not inactivated in the developing lens using this approach (Figure 2B), lens development did not proceed any further and the primordial lens remained attached to the epidermis (Figure 2B, C). Since no lens-associated structures could be detected in mutant embryos at later stages suggests that the lens cells that are initially formed subsequently deteriorates (Figure 1B and C, right panels). These data show that in the mutant embryos the optic vesicle is unable to transform into the optic cup and the lens does not develop.

### Canonical Wnt/β-catenin Signalling is Required for RPE Cell Commitment

**Figure 3:**
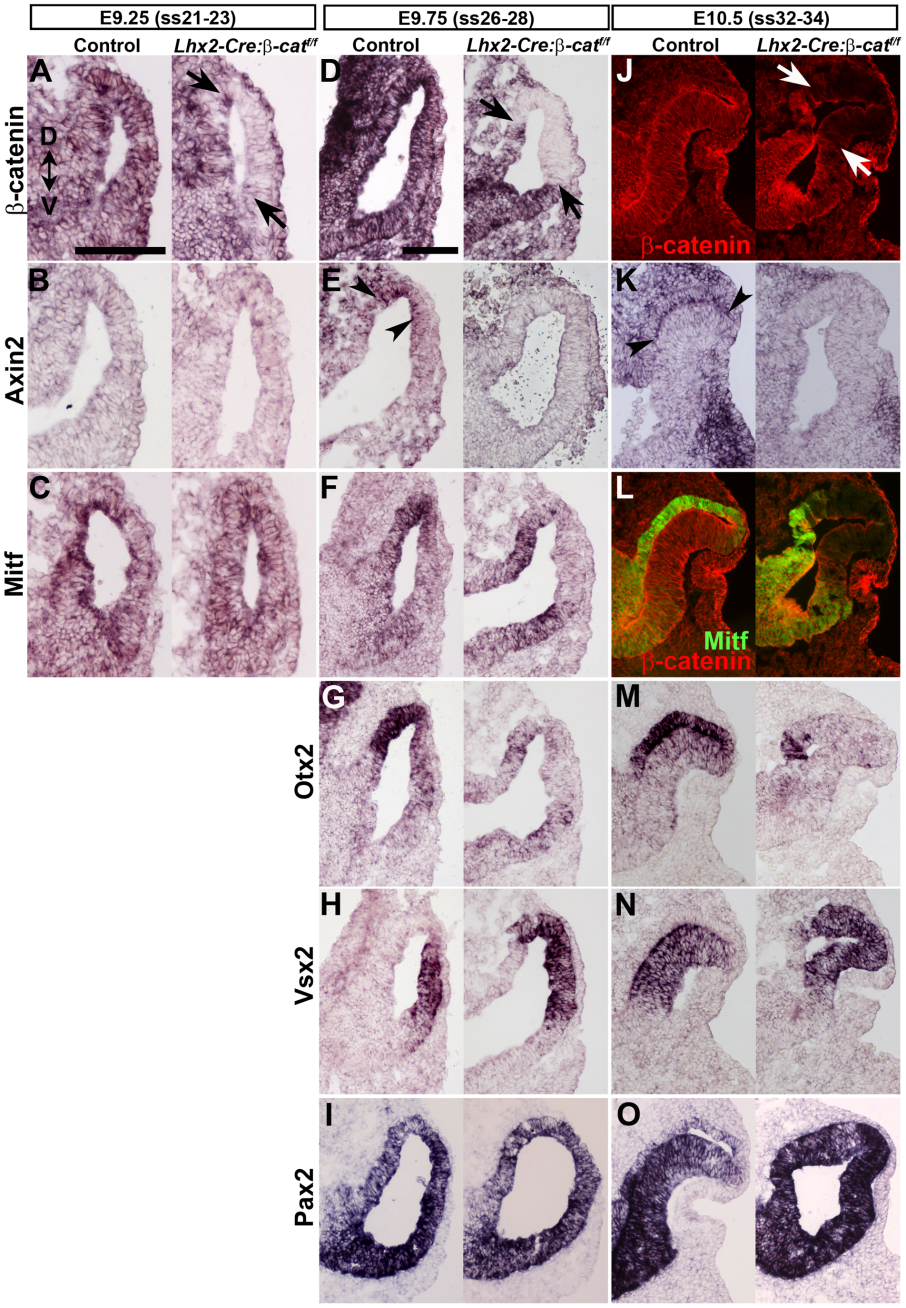
Canonical Wnt/β-catenin signalling is required for RPE cell commitment. (A–C) *In situ* hybridization analyses of the indicated genes on coronal sections of E9.25 somite stage 21–23 (ss21–23) control embryos (left panels) and *Lhx2-Cre:β-catenin^flox/flox^* embryos (right panels). (D–I) *In situ* hybridization analyses of the indicated genes on coronal sections of E9.75 (ss26–28) control embryos (left panels) and *Lhx2-Cre:β-catenin^flox/flox^* embryos (right panels). (J–O) *In situ* hybridization analyses (K, M–O) or immunohistochemical analyses (J, L) for gene expression of the indicated genes on coronal sections of E10.5 (ss32–34) control embryos (left panels) and *Lhx2-Cre:β-catenin^flox/flox^* embryos (right panels). β-catenin protein is indicated by red labelling in J and L while Mitf protein is indicated by green labelling in L. Arrows indicate the area where *β-catenin* has been inactivated in A, D and J. Arrow heads indicate the area of Axin2 expression in E and K. Dorsal-ventral (D–V) orientation for all panels is indicated in A. Scale bars: (A–C and D–O) 100 µm.

Canonical Wnt/β-catenin signalling has been shown to be involved in the maintenance of the RPE layer and to maintain dorsal identity of the optic vesicle [31], [34], [35]. We therefore examined if canonical Wnt signalling was affected in the *β-catenin* mutant optic vesicle by analysing expression of the *Axin2* gene, which is a downstream target of activated canonical Wnt/β-catenin signalling [44], [45]. Our analyses of control embryos showed that *Axin2* expression is not detected at an early stage (E9.25) in the optic vesicle (Figure 3B, left panel). At a slightly later stage (E9.75), *Axin2* is expressed in the dorsal domain of the optic vesicle, both in prospective RPE cells and prospective dorsal neural retina (Figure 3E, left panel). In agreement with previous findings analysing *Axin2* expression or using Wnt/β-catenin signalling reporter mice [34], [35], [46], [47], we find that Axin2 expression becomes restricted to the RPE layer at E10.5 (Figure 3K, left panel). Importantly we find that *Axin2* expression was not detected in the mutant optic vesicle at any stage (Figure 3E, K, right panels), which is consistent with the notion that *β-catenin* is specifically inactivated in this area (Figure 3D, 3J, right panels). To evaluate whether the lack of dorsal Wnt/β-catenin signalling influences the commitment to the various eye compartments, the neural retina, the RPE layer, and the optic stalk, we analysed the expression of genes that delineates these domains. During early development expression of the transcription factor *Mitf* is evenly distributed within the entire optic vesicle, and these cells are thought to represent uncommitted bipotential precursor cells [8]. At this stage inactivation of *β-catenin* had no effect on *Mitf* expression (Figure 3A, 3C), showing that *Mitf* expression in uncommitted precursor cells is independent of Wnt/β-catenin signalling. However, at the time point when *Axin 2* expression was detected (Wnt/β-catenin signalling was activated) in the control optic vesicle, we found that expression of *Mitf* was rapidly down-regulated in the dorsal/distal part of the mutant optic vesicle in the area where *β-catenin* was inactivated (Figure 3F, 3D). At E10.5 Mitf protein becomes undetectable in the area where *β-catenin* is inactivated (Figure 3L, 3J). *Otx2* is also expressed in the prospective RPE cells [10]. Similar to Mitf, Otx2 expression was also rapidly down-regulated in the mutant optic vesicle in the region where *β-catenin* is inactivated at E9.75 (Figure 3G, 3D) and at E10.5 Otx2 expression becomes undetectable in the area where *β-catenin* is inactivated (Figure 3M, 3J). The down-regulated expression of Mitf and Otx2 in the mutant optic vesicle was accompanied by a dorsal expansion of the neural retina-specific gene *Vsx2* (also *Chx10*), whose expression domain appeared to replace that of Mitf and Otx2 expression (Figure 3H, 3N). The optic stalk which is located within the ventral part of the optic vesicle/cup express Pax2 [48], [49]. In control embryos Pax2 is expressed in the entire optic vesicle at E9.75 (Figure 3I) and becomes down-regulated in the RPE layer at E10.5 (Figure 3O, left panel). Since the RPE layer did not form in the mutant the entire optic vesicle structure remained Pax2^+^ at E10.5 (Figure 3O, right panel). These results suggest that a critical step in RPE commitment is that *Mitf* expression transforms from being Wnt/β-catenin-independent to become Wnt/β-catenin-dependent, which extends previously published results showing that β-catenin is important for maintaining RPE cell identity [34], [35].

### Several Changes in Cellular Characteristics Occur in the Mutant Optic Vesicle

**Figure 4:**
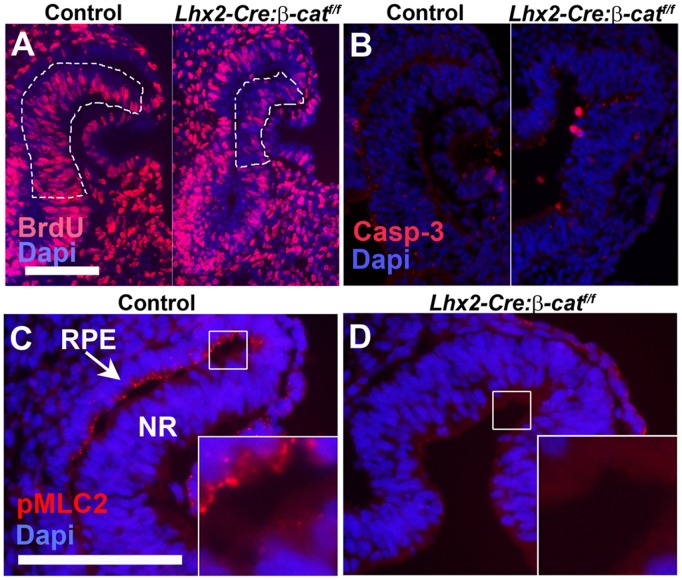
Decreased proliferation and change in physical properties of mutant optic vesicle. (A) Immunohistochemical analysis for the presence of BrdU-labelled cells in the prospective neural retina on a coronal section of an E10.5 control embryo (left panel) and an *Lhx2-Cre:β-catenin^flox/flox^* embryo (right panel). The part of the prospective neural retina that was analysed for BrdU^+^ cells in the control and mutant embryos are outlined. (B) Immunohistochemical analysis for the presence apoptotic cells as revealed by the presence of activated Casp-3^+^ cells on coronal section of an E10.5 control embryo (left panel) and a *Lhx2-Cre:β-catenin^flox/flox^* embryo (right panel). (C) Immunohistochemical analyses for the presence of phosphorylated myosin light chain 2 (pMLC2) on a coronal section of an E10.5 control embryo. (D) Immunohistochemical analyses for the presence of pMLC2 on a coronal section on an E10.5 *Lhx2-Cre:β-catenin^flox/flox^* embryo. Insets in C and D are a magnification of the areas indicated by the squares. Scale bars: (A, B and C, D) 100 µm.

The correct timing of formation of the RPE layer appears to be essential for the expansion of the neural retina. To obtain further molecular insights into how the lack of RPE cell formation affects the development of the neural retina, we initially wanted to elucidate if there is a difference in the number of proliferating cells and/or dying cells in the prospective neural retina in the mutant compared to the control embryos. To address this issue we measured cell proliferation by assessing the relative number of cells incorporating BrdU (i.e. cells in the S-phase of the cell cycle) in the prospective neural retina in the control and mutant embryos at a stage when optic cup formation was initiated. At this stage 57% (±6) of the cells in the control embryos and 34% (±4) of the cells in the mutant embryos were BrdU^+^ (p<0.00001) (Figure 4A), revealing that already at E10.5 the relative number of BrdU^+^ cells was decreased by 40% in the prospective neural retina in the mutant embryos compared to the control embryos. To quantify the number of dying cells we compared the presence of activated Caspase-3 (Casp-3), a marker for apoptotic cells, in the control and mutant embryos at E10.5. There was a slight increase in the number of cells containing activated Casp-3 cells in the mutant neural retina (4.5/section of neural retina ±2.7) compared to control embryos (1.6/section of neural retina ±1.2) (p<0.001) (Figure 4B). Thus, the lack of commitment to RPE cells in the mutant embryos also causes a significant decrease in cell proliferation and some increase in apoptotic cells in the developing neural retina.

It has been suggested that the transition of the optic vesicle to the optic cup is due to a self-organizing mechanism where the RPE layer imposes a structural constraint on the neural retina leading to an apically convex invagination of the neural retina into the RPE layer [50]. This is caused by a difference in mechanical rigidity between the neural retina and the adjacent RPE layer. At the cellular level this results from a high level of phosphorylated myosin light chain 2 (pMLC2) in the RPE cells reflecting local actomyosin activation [51], which is absent in the retinal cells causing a higher mechanical rigidity of the RPE layer compared to the neural retina [4], [50]. Consistent with this we found in the control embryos that pMLC2 level was high in the RPE cells and diminished in the neural retina (Figure 4C). However, in the mutant embryos the pMLC2 level was reduced throughout the entire structure (Figure 4D), consistent with the notion that the mutant optic vesicle adopts a neural retinal fate. This suggests that a critical component of the self-organizing mechanism for optic cup formation is lost in the mutant eye.

### β-catenin is Important for Maintenance of Dorsoventral Patterning of the Retina

**Figure 5:**
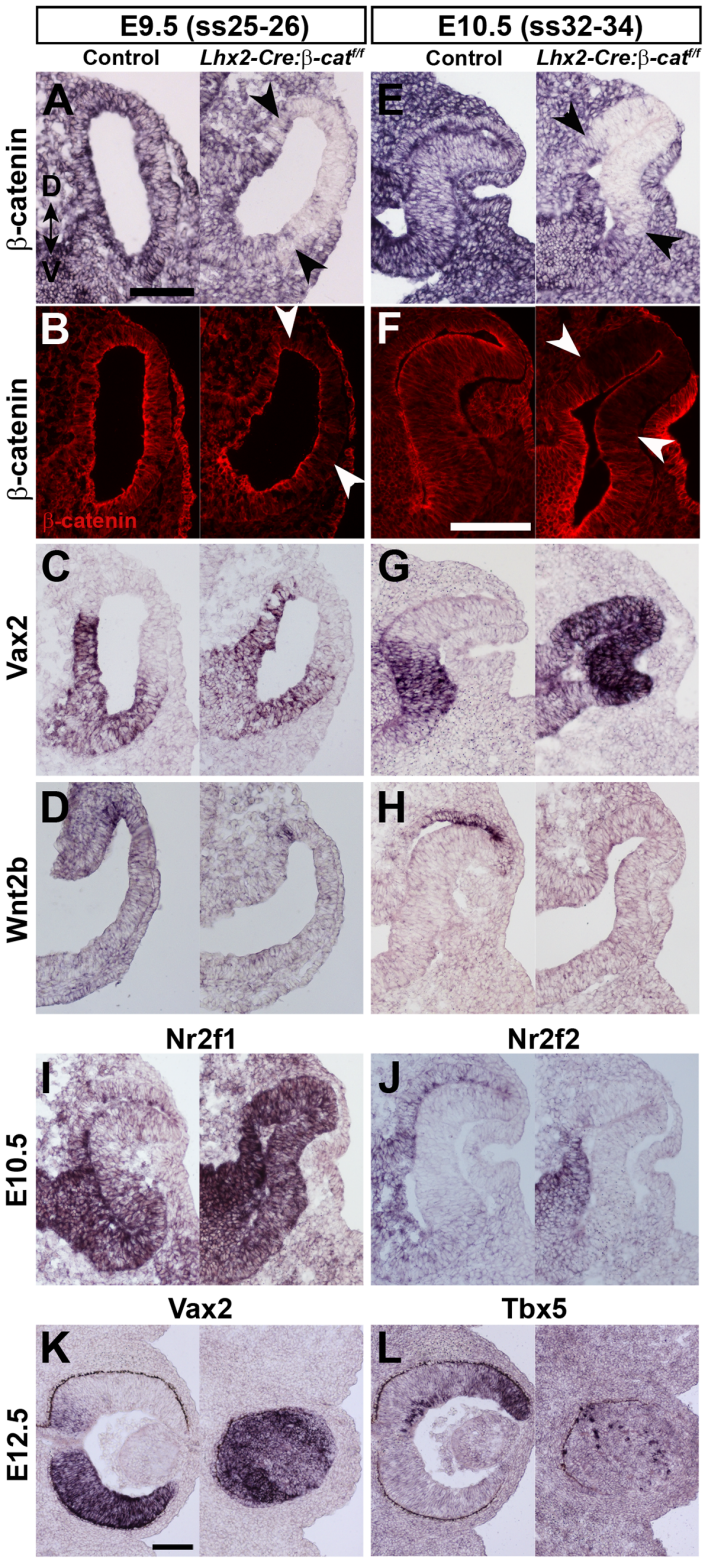
β-catenin is important for maintenance of dorsoventral patterning of the retina. (A–D) *In situ* hybridization analyses (A, C, D) and immunohistochemical analysis (B) on coronal sections of E9.5 (ss25–26) optic vesicles from control (left panels) and on *Lhx2-Cre:β-catenin^flox/flox^* embryos (right panels). (E–H) In situ hybridization analyses (E, G, H) and immunohistochemical analysis (F) on coronal sections of E10.5 (ss32–34) optic cups from control (left panels) and *Lhx2-Cre:β-catenin^flox/flox^* embryos (right panels). Black arrow heads in A and E, and white arrow heads in B and F, indicate the boundaries of the area where β-catenin has been inactivated. (I, J) *In situ* hybridization analyses on coronal sections of E10.5 (ss32–34) optic cups from control (left panel) and *Lhx2-Cre:β-catenin^flox/flox^* embryos (right panels). (K, L) *In situ* hybridization analyses on coronal sections of an eye from E12.5 control (left panels) and *Lhx2-Cre:β-catenin^flox/flox^* embryos. Dorsal-ventral (D–V) orientation of all panels is indicated in A. Scale bars: (A–E, G–J, F and K, L) 100 µm.

To evaluate if lack of Wnt/β-catenin signalling in the dorsal optic vesicle in the β-catenin mutant affects dorsal patterning in general, we analysed the expression of a number of genes known to be involved in dorsal patterning of the optic vesicle. *Bmp4*, *Tbx5* and *Raldh1* are normally expressed in the dorsal domain of the optic vesicle at E9.5 and in the optic cup at E10.5 (Figure S1A–C and S1D–F, left panels). The expression of these genes was attenuated in the mutant optic vesicle at E9.5 (Figure S1A–C, right panels) and by E10.5 expression was either not detected or significantly down-regulated (Figure S1D–F, right panels). To address whether β-catenin is important for initiating dorsal patterning we analysed gene expression of *Bmp4* at an earlier time point. At E9.25 *Bmp4* was clearly expressed in the dorsal part of the optic vesicle although *β-catenin* was inactivated in the mutants (Figure S1G, S1H). This suggests that consistent with previous findings in zebrafish, induction of dorsal patterning is not dependent on Wnt/β-catenin signalling whereas maintenance of dorsal patterning is [31]. The down-regulated expression of dorsal markers in the mutant optic vesicle also leads to an apparent expansion of the expression domain of ventral specific genes, such as *Vax2* (Figure 5C, 5G). Later during embryonic development essentially all cells in the mutant optic rudiment adopt a ventral identity as the remaining cells almost exclusively expressed *Vax2* (Figure 5K) and expression of *Tbx5* was virtually absent (Figure 5L). Wnt2b is a ligand eliciting canonical Wnt signalling that is expressed in the dorsal part of the optic vesicle and in the prospective RPE cell layer area at the stages analysed herein (Figure 5D, 5H) [52], [53]. Expression of *Wnt2b* was down-regulated in the dorsal part of the mutant optic vesicle at E9.5 (Figure 5D), and was undetectable at E10.5 (Figure 5H). Moreover, the transcription factors *Nr2f1* and *Nr2f2* (also *COUP-TF1* and *COUP-TF2*, respectively) have recently been shown to be involved in dorsoventral patterning of the optic vesicle [20], [54]. *Nr2f1* is preferentially expressed in the ventral part and *Nr2f2* is preferentially expressed in the prospective RPE cells in the optic cup (Figure 5I, 5J), but in the mutant optic vesicle we observe that the expression domain of *Nr2f1* is expanded dorsally whereas expression of *Nr2f2* is not detected in this area (Figure 5I, 5J). Therefore, the inital unaffected expression of *Bmp4* in the mutant together with the latter ventralisation and loss of dorsal retinal markers in the optic rudiment, support the conclusion that induction of dorsal identity in the mutant is normal whereas *β-catenin* is essential for maintaining dorsal identity.

### Transcription Factors that are Preferentially Expressed in the Neural Retina become Expressed in the Domain Where the RPE Cells Should Develop

**Figure 6:**
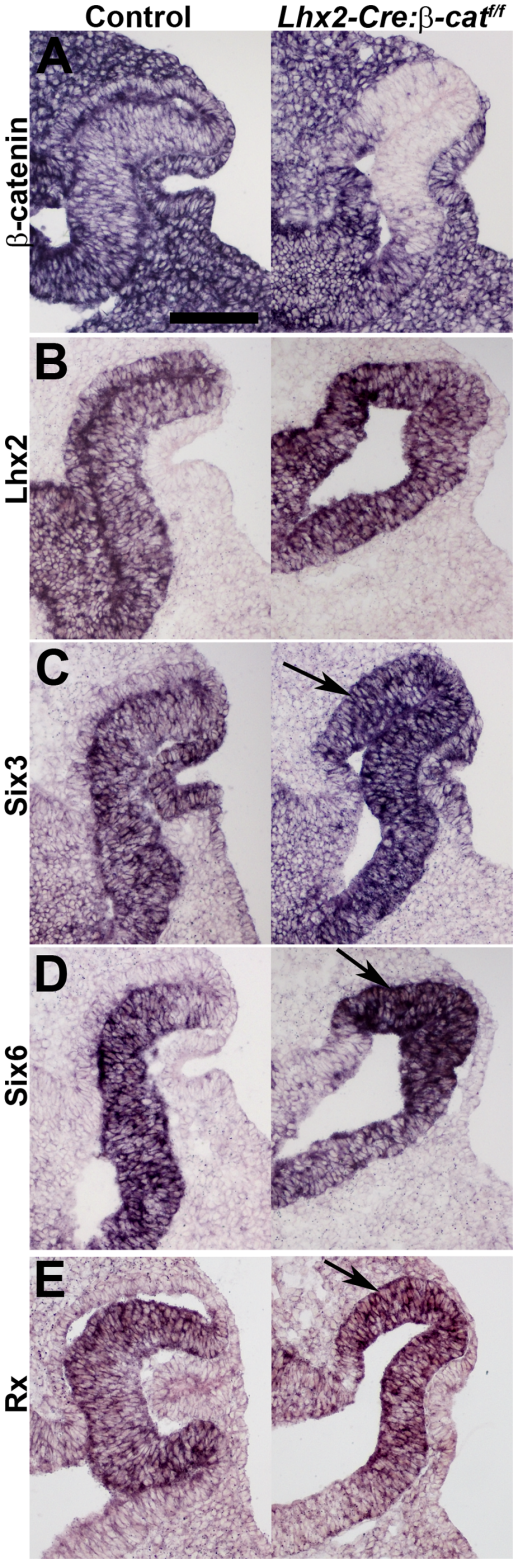
Dorsal shift in expression of retina-specific eye field transcription factors in the mutant optic vesicle. (A–E) *In situ* hybridization analyses on coronal sections of E10.5 optic cups from control embryos (left panels) and *Lhx2-Cre:β-catenin^flox/flox^* mutant embryos (right panels). Arrows indicate the dorsal shift in expression of the retina-specific transcription factors *Six3*, *Six6* and *Rx* in mutant embryos (C–E). Scale bar: 100 µm.

Since lack of dorsal Wnt/β-catenin signalling had such severe effects on eye development, we wanted to determine if expression of transcription factors known to be critical for the progression of eye development were affected in mutant optic vesicles. This includes transcription factors *Lhx2*, *Pax6*, *Six3*, *Six6* and *Rx*. *Lhx2* and *Pax6* are ubiquitously expressed in the control and mutant optic vesicle (Figure 6B and 2C). However, expression of *Six3*, *Six6* and *Rx* that ultimately becomes restricted to the neural retina in the controls fail to become restricted in the mutants, but instead expand into the region where RPE cells should develop (Figure 6C–E). Thus, these findings are in agreement with our previous data suggesting that the cells that normally adopt an RPE cell fate instead become committed to neural retina fate in the mutant optic vesicle/cup. This study complements previous results when *β-catenin* is inactivated in the RPE layer [34], [35], since further development of any organised eye structure is completely blocked in the *β-catenin* mutant embryos presented in this study.

### β-catenin Remains Associated with N-cadherin and F-actin at the Apical Side of the Cells in the Mutant Optic Vesicle

**Figure 7:**
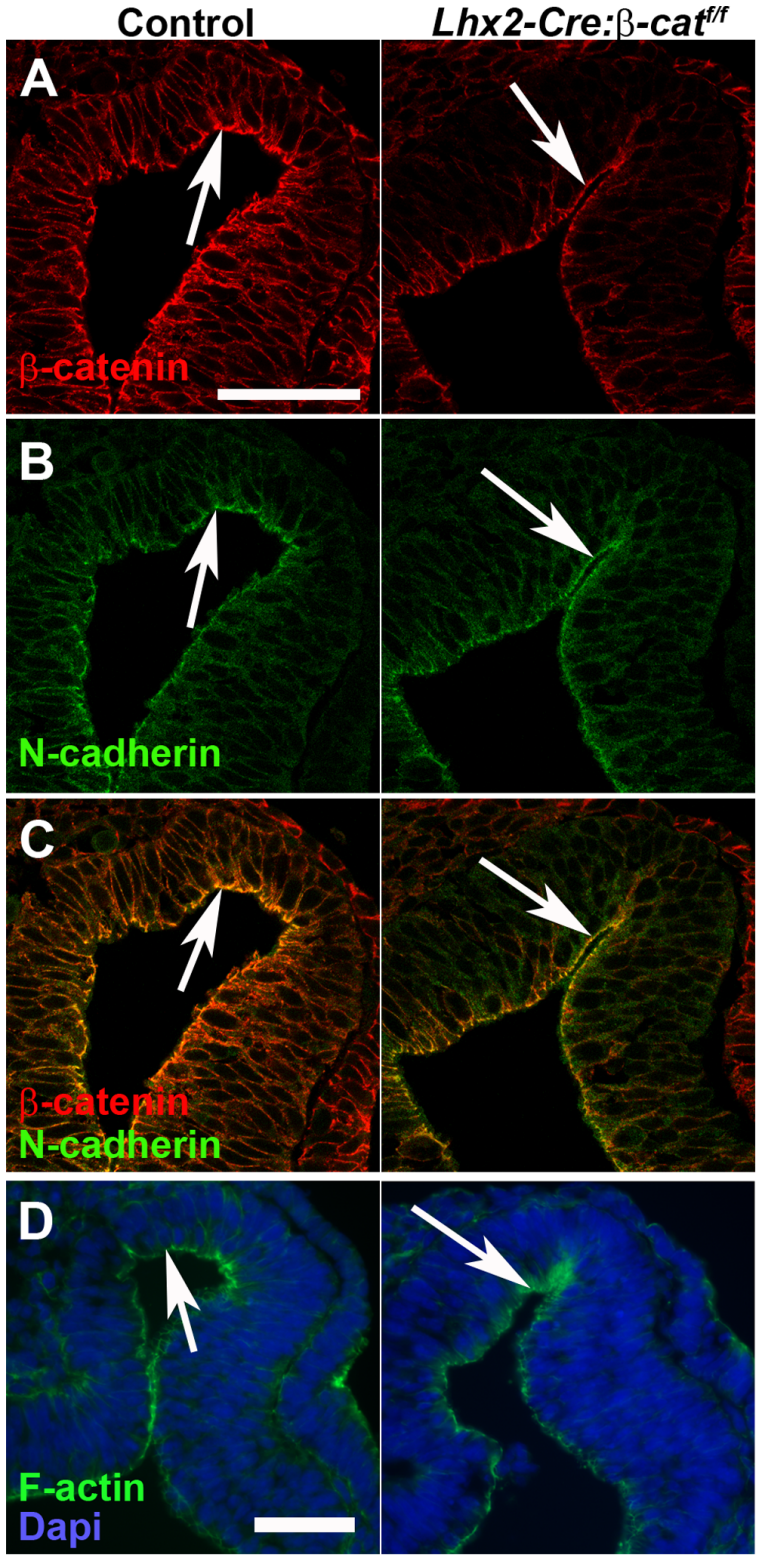
β-catenin remains associated with N-cadherin and F-actin at the apical side of the cells in the mutant optic vesicle. (A–D) Immunohistochemical analyses for cellular localisation of the indicated proteins on coronal sections of E10 (ss30–31) optic vesicles from control embryos (left panels) and *Lhx2-Cre:β-catenin^flox/flox^* embryos (right panels). (C) is a merge of (A) and (B) to show co-localisation (yellow) in the apical side of the cells in the optic vesicle on both control and mutant embryos (arrows). (D) A serial section of the same embryo as in (A–C) revealing F-actin protein at the apical side of the cells in both control and mutant optic vesicles (arrows). Scale bars: (A–C and D) 50 µm.

In addition to mediating canonical Wnt signalling, β-catenin has also been shown to be involved in cell-cell adhesion since it interacts with the cytoplasmic part of cadherins and links it to actin filaments (F-actin) in the cytoskeleton [27]. To examine if this function of β-catenin is affected in the mutant embryos, we analysed the cellular distribution of β-catenin, N-cadherin and F-actin in the late optic vesicle/early optic cup. In the control optic vesicle, β-catenin, N-cadherin and F-actin are associated with each other at the cell membrane on the apical side of the cells in the optic vesicle (Figure 7A–D, left panels). In the mutant embryos, β-catenin remains associated with N-cadherin and F-actin at the apical membrane at this early stage (Figure 7A–D, right panels. See also Figure 5A, 5B, 5E and 5F for the distribution of cells with inactivated β-catenin mRNA and decreased levels of β-catenin protein in the optic vesicle). Also at the later stage of development detectable levels of β-catenin remains associated with N-cadherin and F-actin at the cell membrane (E10.5, Figure S2). Thus, although the non-N-cadherin-associated β-catenin is rapidly depleted in the β-catenin-negative part of the mutant optic vesicle, the apical β-catenin appears to be more stable and remains associated with N-cadherin and F-actin for some time. Although we cannot exclude that reduced levels of N-cadherin-associated β-catenin might contribute to the phenotype, the maintained association of β-catenin and N-cadherin at the apical side of the cells suggest that this cellular function of β-catenin may not be affected to any major extent at the early stages of optic vesicle to optic cup transformation.

### A Few RPE Cells can Escape β-catenin Inactivation Leading to the Formation of an Optic Rudiment

**Figure 8:**
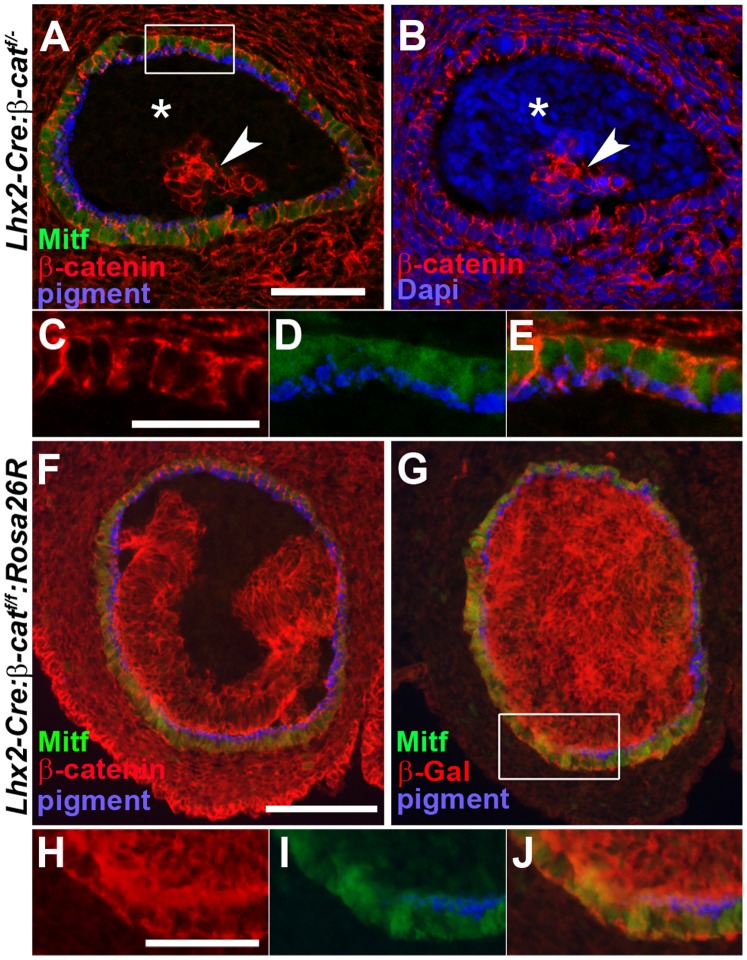
The few RPE cells that develop in the mutant embryos are derived from eye committed progenitor cells in the anterior neural plate that escape *β-catenin* inactivation. (A) Immunohistochemical analyses of Mitf (green) and β-catenin (red) on coronal sections of an E13.5 optic rudiment in an *Lhx2-Cre:β-catenin^flox/^* ^−^ embryo. RPE cells are identified by pigment (pseudocoloured blue) and Mitf (green). (B) Immunohistochemical analysis of β-catenin and DAPI labelling of the same coronal section as in (A) to reveal that both β-catenin^+^ and β-catenin^−^ cells are present in the optic rudiment that develops in mutant embryos. * indicates the area of β-catenin^−^ non-RPE cells and the arrow head indicates the area of β-catenin^+^ non-RPE cells in the optic rudiment. (C–E) is a magnification of the area indicated by a rectangle in (A). (C) and (D) are merged in (E) to show co-localisation of β-catenin, Mitf and pigment. (F) Immunohistochemical analysis of Mitf (green) and β-catenin (red) on a coronal section of an E12.5 optic rudiment in a *Lhx2-Cre:β-catenin^flox/flox^:ROSA26R* embryo. (G) Immunohistochemical analysis of Mitf (green) and β-Gal (red) on a serial section following (F) revealing that all cells in the optic rudiment, including the RPE cells, are β-Gal^+^ and hence derived from Cre^+^ progenitor cells in the anterior neural plate. (H–J) is a magnification of the area indicated by a rectangle in (G). (H) and (I) are merged in (J) to show co-localisation of Mitf, β-Gal and pigment. Scale bars: (A, B, and H–J) 50 µm. (C–E) 25 µm. (F, G) 100 µm.

Since our results suggest that formation of the RPE layer is dependent on active canonical Wnt/β-catenin signalling it was surprising that a small number of RPE cells developed in most of the mutant embryos (Figure 1B, 1C). However, all mutant embryos analysed (n = 4, both eyes) revealed that the few RPE cells that did develop always expressed β-catenin protein (Figure 8A–F), supporting the conclusion that formation of RPE cells is dependent on functional β-catenin expression. The non-RPE cells in the optic rudiment that developed in the mutant embryos were both β-catenin^+^ and β-catenin^−^ (Figure 8A, 8B), revealing that these cells could develop in a β-catenin-independent manner but consistently became ventralised (Figure 5K, 5L). Of particular note, all the mutant embryos analysed where an optic rudiment could be observed contained RPE cells (n = 19) (Figure 8A, 8B and Figure 1B, 1C). This suggests that maintenance of the non-RPE cells is dependent on the formation of RPE cells. We have previously shown that all of the cells derived from the optic vesicle (neural retina, optic stalk and RPE cells) are progeny of the cells expressing the Cre recombinase in the Lhx2-Cre mouse line since all these cell types were lineage traced when the *Lhx2-Cre* mouse strain was crossed to the ROSA26 reporter (*ROSA26R*) mouse [37]. Given that a few cells in the optic rudiment expressed β-catenin, we wanted to confirm that the cells escaping *β-catenin* inactivation were derived from the progenitor cells defined by Cre expression and not from a separate progenitor cell population in the anterior neural plate. To distinguish between these two possibilities, we crossed the *Lhx2-Cre*, *β-catenin^flox/flox^* and *ROSA26R* mouse lines to generate *Lhx2-Cre:β-catenin^flox/flox^:ROSA26R* mouse embryos. Since all lineage-traced cells express β-Galactosidase (β-Gal) we analysed the mutant embryos for β-Gal and β-catenin expression. All cells in the mutant optic rudiment, including both the β-catenin^+^ and the β-catenin^−^ cells, were β-Gal^+^ (n = 3, both eyes) (Figure 8F–J). These data strongly suggest that floxed *β-catenin* alleles can escape inactivation even though they originate from the Cre^+^ progenitor cells in the anterior neural plate. Since this observation suggests that Cre-mediated recombination of the *β-catenin* locus is less efficient compared to the *ROSA26R* locus it could explain why the phenotype is consistently more severe in the mouse strain where only one allele of β-catenin has to be inactivated compared to two alleles (*Lhx2-Cre:β-catenin^flox/^* ^−^ vs. *Lhx2-Cre:β-catenin^flox/flox^* embryos) to generate β-catenin^−^ cells (Figure 1B–D). Furthermore, these data also suggest that the size of the optic rudiment correlates to the number of RPE cells that escape β-catenin inactivation.

### Development of Retinal Cell Classes is Independent of β-catenin

As most transcription factors known to be important for eye development were expressed in the mutant optic vesicle, we next wanted to evaluate whether differentiation into more mature cell types occurred in the mutant embryos. We therefore assayed for the presence of different neuronal cell types that normally are located in the various cell layers of the retina. By using markers for more mature committed cells, it appears as if retinal ganglion cells (Brn3^+^) (Figure S3B) [55] rods (Nrl^+^, Crx^+^) (Figure S3C, S3D) [56], [57], cones (Trβ2^+^, Crx^+^) (Figure S3D, S3E) [57], [58], horizontal cells and amacrine cells (Prox1, Ptf1a) [59], [60], [61] could develop (Figure S3F, S3G). Thus, most cell types within the mature neural retina were present in the mutants, although in an apparent random distribution and reduced number. Moreover, these cell types appear to develop from precursor cells located in both β-catenin^+^ as well as β-catenin^−^ areas in the mutant optic rudiment (Figure S3B–G), further supporting the view that the neural cell types in the retina can develop independent of β-catenin [36]. Thus, while some mature neurons develop, the stereotyped organisation that underlies the topography and function of the normal eye is profoundly disrupted in the mutants.

## Discussion

By inactivating the *β-catenin* gene prior to the cellular commitment into prospective neural retinal cells and RPE cells in the optic vesicle, we have demonstrated that this gene is essential for optic cup formation. β-catenin has at least two different cellular functions; i) activating canonical Wnt signalling by translocation into the nucleus. ii) regulating cell adhesion by binding to the cadherin family of adhesion molecules [27]. We have shown that *β-catenin* mutant embryos have significantly down-regulated canonical Wnt signalling in the dorsal optic vesicle. This reduction in canonical Wnt signalling in the dorsal optic vesicle causes a severely compromised commitment to RPE cells in the mutant embryos as evidenced by a lack of Mitf and Otx2 expression. However, a small number of RPE cells could develop in the mutant embryos leading to the formation of an optic rudiment. While we demonstrate that the RPE cells are derived from the Cre^+^ eye committed progenitor cells in the anterior neural plate, we clearly demonstrate that these cells have escaped *β-catenin* inactivation and are hence β-catenin^+^. Moreover, the significantly reduced expression levels of dorsal markers led to an almost complete ventralisation of the non-RPE cells in the optic rudiment. Moreover, active Wnt/β-catenin signalling in RPE cells at an early stage of optic vesicle development is evolutionary conserved in fish, chicken and frogs [31], [52], [62], [63]. Collectively, our and other observations strongly suggest that the severe eye phenotype observed in the *β-catenin* mutant embryos is due to the lack of canonical Wnt signalling resulting in blockade of RPE cell commitment in the optic vesicle. RPE cells have been suggested to be specified by the TGFβ family member Activin A which is secreted by the extraocular mesenchyme [6]. Whether TGFβ signalling is upstream of Wnt signalling or if they act in parallel to induce commitment to RPE remains to be elucidated.

In previous studies β-catenin was inactivated during eye development at a stage when the optic cup had been formed [34], [35], hence the RPE and the neural retina were already specified in these studies. β-catenin was inactivated in the RPE cell layer which lead to the loss of RPE cell identity and transdifferentiation of the committed RPE cells into neural retinal cells [34], [35]. A similar phenotype is also observed when the transcription factors *Mitf* or *Otx2* are inactivated, or when both *Nr2f1* and *Nr2f2* are inactivated [8], [10], [54], [64]. β-catenin has been suggested to be important for maintaining RPE cell identity by directly binding to putative Tcf/Lef binding sites in the *Mitf* and *Otx2* enhancers [35]. Moreover, in the previous studies where β-catenin was inactivated during eye development the mutant eye develops relatively mature structures including a lens and neural retina, revealing that the phenotype is considerably milder compared to the phenotype described in this study. It has also been shown that β-catenin and Lef1 can directly interact with Mitf to induce Mitf-specific target genes in melanocytes [65], [66], indicating that Wnt/β-catenin signalling is important for maintenance of pigmented cells. Whether this is the case for RPE cells remains to be determined. However, our study extends these findings by suggesting that β-catenin is also essential for the specification of the RPE cells, which is essential for optic cup formation. We show here that β-catenin inactivation prior to the specification of the RPE and the retina extends the Vsx2^+^ neural retina domain at the expense of the Mitf^+^/Otx2^+^ RPE domain. This change in fate is further supported by the expression of the retina-specific genes *Six6*, *Six3* and *Rx* that appears to replace expression of the RPE-specific genes *Otx2*, *Wnt2b* and *Nr2f2*. Although delayed, the subsequent transition of the optic vesicle to the optic cup is initiated in the mutant embryos as the optic vesicle invaginates, leading to the formation of a two-layered structure and a simultaneous invagination of the lens placode. At this stage eye morphogenesis is developmentally arrested in the mutant embryos and an optic cup is never formed. This is most likely due to that the mutant optic vesicle is unable to form an RPE layer at the correct time. Thus, the eye phenotype in the *Lhx2-Cre:β-catenin^flox/^* ^−^ and *Lhx2-Cre:β-catenin^flox/flox^* embryos we observe here is much more severe compared with the earlier studies on *β-catenin* inactivation during eye development. The results presented in this study are reminiscent of the eye phenotype observed when the development of RPE cells are severely compromised using another approach [67]. Together, this suggests that specification and expansion of RPE cells at the correct time are essential steps in the development of the eye during optic vesicle to optic cup transformation. The inability to form the optic cup when the RPE layer formation is severely compromised is in agreement with the “Relaxation-expansion model” for self-driven morphogenesis [50]. This model predicts that the formation of an optic cup is based on the premise that the retina decreases its rigidity during its specification. This is thought to be achieved by down-regulation of the microtubule coordinator pMLC2 levels in the retina compared with the RPE layer which maintains high levels of pMLC2. This results in the retina expanding inside the less flexible RPE layer [4], [50]. Thus, disruption of the RPE layer and therefore loss of the more rigid “RPE frame” for the retina to fold against means that the optic cup cannot form. Our results suggest that in the mutants since most cells in the optic cup become committed to retinal fate, the whole optic vesicle down-regulates the level of pMLC2. This general down-regulation of pMLC2 presumably leads to a lack of differential rigidity and hence causes it to collapse and thereby preventing the formation of the optic cup. It has recently been suggested that N-cadherin/β-catenin and F-actin interact with MLC [68]. However, since there is no obvious difference in the levels of β-catenin between the neural retina and the RPE cells during normal eye development, pMLC is probably regulated by other cell type specific mechanisms and not directly by β-catenin. Furthermore, the frequency of proliferating cells in the mutant neural retina is significantly reduced and the number of dying cells is slightly increased in the mutant neural retina compared to the control neural retina, suggesting that the RPE cells regulate cell proliferation and to some extent also cell survival in the neural retina in a cell nonautonomous manner. We therefore suggest that the reduced proliferation together with the lack of structural constraint imposed by the RPE cells on the neural retina in the mutant embryos are the major causes of the observed anophthalmia. Most studies conducted thus far studying the supportive role of the RPE layer have addressed its role in regulating photoreceptor survival and differentiation [7]. If these factors overlap with or are distinct from those responsible for regulating the expansion and organisation of the neural retina during early eye development remains to be elucidated.

Although our data strongly suggests that β-catenin is essential for the formation of the RPE layer, a small number of RPE cells were still present in all optic rudiments analysed in the mutants at later developmental stages. However, all the RPE cells that developed in the mutant were β-catenin^+^ and had thus escaped *β-catenin* inactivation, emphasising that β-catenin is essential for RPE cell development. It has been suggested that dorsal and ventral RPE cells develop by different mechanisms, and that development of ventral RPE cells might be independent of β-catenin [12], [34]. Our data suggest that all the RPE cells require β-catenin for their development, irrespective of their dorsoventral origin. In contrast to the RPE cells, the non-RPE cells that developed in the mutant embryos could be either β-catenin^+^ or β-catenin^−^. These non-RPE cells did not form an organised retina although various neurons developed suggesting that commitment of neuronal cell types is not solely dependent on β-catenin and tissue organisation. Moreover, our data implies that in the mutant, non-RPE cells are not maintained in the absence of RPE cells, further arguing for a central role of the RPE cells in the development of the eye.

The data presented here suggest that Wnt/β-catenin signalling is important for maintaining dorsal identity of the retina. Previous studies in zebrafish have also suggested that Wnt/β-catenin signalling is important for maintaining dorsal identity of the retina in a cell nonautonomous manner since Wnt/β-catenin signalling is restricted to the RPE cells in zebrafish [31]. However, we provide evidence that Wnt/β-catenin signalling is active in both prospective RPE cells and prospective dorsal retina, suggesting that the dorsalising effect on the retina mediated by β-catenin is cell autonomous. Wnt2b, a ligand for the canonical Wnt/β-catenin signalling pathway, is expressed in the dorsal part of the optic vesicle/cup and the RPE cells [69], and this gene is down-regulated in our β-catenin mutant suggesting a positive feedback regulation. It should be noted that additional canonical Wnt ligands are probably required for the RPE cell layer formation and dorsalisation of the neural retina since *Wnt2b* null mice have no reported eye defects [70].

Another mediator of canonical Wnt signalling is the Fz co-receptor Lrp6. *Lrp6* ^−*/*−^ mice do share some of the phenotypes with the *β-catenin* mutants presented here, but these are less severe. Although the *Lrp6* ^−*/*−^ mice have microphthalmia with coloboma, they develop the basic structures of the eye: neural retina, RPE cell layer and the lens [32], [71], which is in contrast to the *β-catenin* mutants presented in this work. However, in agreement with our data the neural retina in the *Lrp6* ^−*/*−^ mice lose or down-regulate expression of dorsal markers such as Tbx5, Raldh1 and BMP4 and the expression domain of the ventral marker Vax2 extends dorsally [32], [71]. Since canonical Wnt/β-catenin signalling is suggested to be mediated via Lrp5 or Lrp6 [72], [73], [74], it is possible that Lrp5 might compensate for Lrp6 in the *Lrp6* ^−*/*−^ mice and hence the phenotype will not be as severe as in the *β-catenin* mutant which presumably block all canonical Wnt signalling. Other mediators of Wnt signalling that have been studied during eye development are the Fz receptors Fz5 and Fz8. The *Fz5* ^−*/*−^ *:Fz8^+/^* ^−^ compound mutants show a severe retinal coloboma and microphthalmia although the major eye structures, neural retina, RPE cells and lens, developed in this mutant [75]. Since there are a number of Fz receptor expressed in the eye during development [76], it is highly likely that other Fz receptors could compensate for loss of Fz5 expression and decrease in Fz8 expression and hence ameliorate the phenotype compared to the *β-catenin* mutant mice presented in our study.

In conclusion, β-catenin is important for eye development since it is essential for the transition of the optic vesicle to the optic cup. This defect is due to the fact that commitment of RPE cells depends on β-catenin. Moreover, β-catenin is also important for the dorsoventral patterning of the retina whereas differentiation of the various cells in the retina is independent of β-catenin.

## Supporting Information

Figure S1 **Maintenance but not induction of dorsal identity of the optic vesicle is dependent on β-catenin.** (A–C) *In situ* hybridization analyses on coronal sections of E9.5 (ss25–26) optic vesicles from control (left panels) and *Lhx2-Cre:β-catenin^flox/flox^* embryos (right panels). (D–F) In situ hybridization analyses on coronal sections of E10.5 (ss32–34) optic cups from control (left panels) and *Lhx2-Cre:β-catenin^flox/flox^* embryos (right panels). (G, H) *In situ* hybridization analyses on coronal sections of E9.25 (ss21–23) optic vesicles from control embryos (left panels) and *Lhx2-Cre:β-catenin^flox/flox^* embryos (right panels). Arrow heads indicate the boundaries where β-catenin has been inactivated in the optic vesicle. Arrows indicate Bmp4 expression. Dorsal-ventral (D–V) orientation for all panels is indicated in A. Scale bars: (A–F and G, H) 100 µm.
(TIF)

Figure S2 **β-catenin protein remains associated with N-cadherin and F-actin in the mutant optic vesicle at E10.5.** (A–D) Immunohistochemical analyses for cellular localisation of the indicated proteins on coronal sections of E10.5 (ss 33–35) optic vesicles from control embryos (left panels) and *Lhx2-Cre:β-catenin^flox/flox^* embryos (right panels). (A) and (B) are merged in (C) to show co-localisation (yellow) on the apical side of the cells in the optic vesicle. (D) Serial section following those in (A–C). Note that while the RPE and the neural retina have been specified in the control embryo (left panels), the corresponding structure in the mutant embryo contains almost exclusively cells of neural retinal fate at this developmental stage (see Figure 3L, 3M, and 3N). Scale bar: 100 µm.
(TIF)

Figure S3 **Retinal cell classes can develop independent of β-catenin.** (A) Hematoxylin/eosin staining of coronal sections of an E18.5 control embryo (left panel) and an *Lhx2-Cre:β-catenin^flox/flox^* mutant embryo (right panel). Arrow indicates the optic rudiment that can develop in mutant embryos (right panel). (B, F, G) Immunohistochemical analyses for the presence of the indicated proteins on coronal sections of control (left panels) and mutant embryos (right panels). (C–E) *In situ* hybridization analyses for gene expression analysis of the indicated genes on coronal sections of control (left panels) and mutant embryos (right panels). All the sections from the mutant embryo have been analysed for β-catenin expression on a consecutive section to ensure that the distribution of β-catenin^+^ and β-catenin^−^ cells shown in panel (B), is maintained in all panels. Scale bars: (A) 500 µm. (B–G, left and right panels) 100 µm.
(TIF)

## References

[1] Wilson SW, Houart C (2004) Early steps in the development of the forebrain. Dev Cell 6: 167–181. 14960272 10.1016/s1534-5807(04)00027-9 PMC2789258

[2] Kaufman M (1994) The atlas of mouse development. San Diego: Academic Press Limited.

[3] Chow RL, Lang RA (2001) Early eye development in vertebrates. Annu Rev Cell Dev Biol 17: 255–296. 11687490 10.1146/annurev.cellbio.17.1.255

[4] Eiraku M, Takata N, Ishibashi H, Kawada M, Sakakura E, et al (2011) Self-organizing optic-cup morphogenesis in three-dimensional culture. Nature 472: 51–56. 21475194 10.1038/nature09941

[5] Horsford DJ, Nguyen MT, Sellar GC, Kothary R, Arnheiter H, et al (2005) Chx10 repression of Mitf is required for the maintenance of mammalian neuroretinal identity. Development 132: 177–187. 15576400 10.1242/dev.01571

[6] Fuhrmann S, Levine EM, Reh TA (2000) Extraocular mesenchyme patterns the optic vesicle during early eye development in the embryonic chick. Development 127: 4599–4609. 11023863 10.1242/dev.127.21.4599

[7] Strauss O (2005) The retinal pigment epithelium in visual function. Physiol Rev 85: 845–881. 15987797 10.1152/physrev.00021.2004

[8] Nguyen M-TT, Arnheiter H (2000) Signaling and transcriptional regulation in early mammalian eye development: a link between FGF and MITF. Development 127: 3581–3591. 10903182 10.1242/dev.127.16.3581

[9] Bovolenta P, Mallamaci A, Briata P, Corte G, Boncinelli E (1997) Implication of OTX2 in pigment epithelium determination and neural retina differentiation. J Neurosci 17: 4243–4252. 9151741 10.1523/JNEUROSCI.17-11-04243.1997 PMC6573571

[10] Martinez-Morales JR, Signore M, Acampora D, Simeone A, Bovolenta P (2001) Otx genes are required for tissue specification in the developing eye. Development 128: 2019–2030. 11493524 10.1242/dev.128.11.2019

[11] Nishida A, Furukawa A, Koike C, Tano Y, Aizawa S, et al (2003) Otx2 homeobox gene controls retinal photoreceptor cell fate and pineal gland development. Nat Neurosci 6: 1255–1263. 14625556 10.1038/nn1155

[12] Zhang XM, Yang XJ (2001) Temporal and spatial effects of Sonic hedgehog signaling in chick eye morphogenesis. Dev Biol 233: 271–290. 11336495 10.1006/dbio.2000.0195 PMC7048387

[13] Dudley AT, Lyons KM, Robertson EJ (1995) A requirement for bone morphogenetic protein-7 during development of the mammalian kidney and eye. Genes Dev 9: 2795–2807. 7590254 10.1101/gad.9.22.2795

[14] Luo G, Hofmann C, Bronckers AL, Sohocki M, Bradley A, et al (1995) BMP-7 is an inducer of nephrogenesis, and is also required for eye development and skeletal patterning. Genes Dev 9: 2808–2820. 7590255 10.1101/gad.9.22.2808

[15] Furuta Y, Hogan BL (1998) BMP4 is essential for lens induction in the mouse embryo. Genes Dev 12: 3764–3775. 9851982 10.1101/gad.12.23.3764 PMC317259

[16] Wawersik S, Purcell P, Rauchman M, Dudley AT, Robertson EJ, et al (1999) BMP7 acts in murine lens placode development. Dev Biol 207: 176–188. 10049573 10.1006/dbio.1998.9153

[17] Müller F, Rohrer H, Vogel-Höpker A (2007) Bone morphogenetic proteins specify the retinal pigment epithelium in the chick embryo. Development 134: 3483–3493. 17728349 10.1242/dev.02884

[18] Behesti H, Holt JK, Sowden JC (2006) The level of BMP4 signaling is critical for the regulation of distinct T-box gene expression domains and growth along the dorso-ventral axis of the optic cup. BMC Dev Biol 6: 62. 17173667 10.1186/1471-213X-6-62 PMC1764729

[19] Koshiba-Takeuchi K, Takeuchi JK, Matsumoto K, Momose T, Uno K, et al (2000) Tbx5 and the retinotectum projection. Science 287: 134–137. 10615048 10.1126/science.287.5450.134

[20] Satoh S, Tang K, Iida A, Inoue M, Kodama T, et al (2009) The spatial patterning of mouse cone opsin expression is regulated by bone morphogenetic protein signaling through downstream effector COUP-TF nuclear receptors. J Neurosci 29: 12401–12411. 19812316 10.1523/JNEUROSCI.0951-09.2009 PMC2791207

[21] Sanes JR, Zipursky SL (2010) Design principles of insect and vertebrate visual systems. Neuron 66: 15–36. 20399726 10.1016/j.neuron.2010.01.018 PMC2871012

[22] Cepko CL, Austin CP, Yang X, Alexiades M, Ezzeddine D (1996) Cell fate determination in the vertebrate retina. Proc Natl Acad Sci USA 93: 589–595. 8570600 10.1073/pnas.93.2.589 PMC40096

[23] La Vail MM, Rapaport DH, Rakic P (1991) Cytogenesis in the monkey retina. J Comp Neurol 309: 86–114. 1894769 10.1002/cne.903090107

[24] Stiemke MM, Hollyfield JG (1995) Cell birthdays in Xenopus laevis retina. Differentiation 58: 189–193. 7713326 10.1046/j.1432-0436.1995.5830189.x

[25] Young RW (1985) Cell differentiation in the retina of the mouse. Anat Rec 212: 199–205. 3842042 10.1002/ar.1092120215

[26] Carter-Dawson LD, LaVail MM (1979) Rods and cones in the mouse retina. II. Autoradiographic analysis of cell generation using tritiated thymidine. J Comp Neurol 188: 263–272. 500859 10.1002/cne.901880205

[27] Valenta T, Hausmann G, Basler K (2012) The many faces and functions of β-catenin. EMBO J 31: 2714–2736. 22617422 10.1038/emboj.2012.150 PMC3380220

[28] Aberle H, Bauer A, Stappert J, Kispert A, Kemler R (1997) β-catenin is a target for the ubiquitin-proteasome pathway. EMBO J 16: 3797–3804. 9233789 10.1093/emboj/16.13.3797 PMC1170003

[29] Zeng X, Huang H, Tamai K, Zhang X, Harada Y, et al (2008) Initiation of Wnt signaling: control of Wnt coreceptor Lrp6 phosphorylation/activation via frizzled, dishevelled and axin functions. Development 135: 367–375. 18077588 10.1242/dev.013540 PMC5328672

[30] Daniels DL, Weis WI (2005) β-catenin directly displaces Groucho/TLE repressors from Tcf/Lef in Wnt-mediated transcription activation. Nat Struct Mol Biol 12: 364–371. 15768032 10.1038/nsmb912

[31] Veien ES, Rosenthal JS, Kruse-Bend RC, Chien CB, Dorsky RI (2008) Canonical Wnt signaling is required for the maintenance of dorsal retinal identity. Development 135: 4101–4111. 19004855 10.1242/dev.027367 PMC2667153

[32] Zhou CJ, Molotkov A, Song L, Li Y, Pleasure DE, et al (2008) Ocular coloboma and dorsoventral neuroretinal patterning defects in Lrp6 mutant eyes. Dev Dyn 237: 3681–3689. 18985738 10.1002/dvdy.21770 PMC2727282

[33] Maretto S, Cordenonsi M, Dupont S, Braghetta P, Broccoli V, et al (2003) Mapping Wnt/β-catenin signaling during mouse development and in colorectal tumors. Proc Natl Acad Sci USA 100: 3299–3304. 12626757 10.1073/pnas.0434590100 PMC152286

[34] Fujimura N, Taketo MM, Mori M, Korinek V, Kozmik Z (2009) Spatial and temporal regulation of Wnt/beta-catenin signaling is essential for development of the retinal pigment epithelium. Dev Biol 334: 31–45. 19596317 10.1016/j.ydbio.2009.07.002

[35] Westenskow P, Piccolo S, Fuhrmann S (2009) β-catenin controls differentiation of the retinal pigment epithelium in the mouse optic cup by regulating Mitf and Otx2 expression. Development 136: 2505–2510. 19553286 10.1242/dev.032136 PMC2709060

[36] Fu X, Sun H, Klein WH, Mu X (2006) β-catenin is essential for lamination but not neurogenesis in mouse retinal development. Dev Biol 299: 424–437. 16959241 10.1016/j.ydbio.2006.08.015 PMC3385515

[37] Hägglund A-C, Dahl L, Carlsson L (2011) Lhx2 is required for patterning and expansion of a distinct progenitor cell population committed to eye development. PLoS ONE 6: e23387. 21886788 10.1371/journal.pone.0023387 PMC3158764

[38] Brault V, Moore R, Kutsch S, Ishibashi M, Rowitch DH, et al (2001) Inactivation of the β-catenin gene by Wnt1-Cre-mediated deletion results in dramatic brain malformation and failure of craniofacial development. Development 128: 1253–1264. 11262227 10.1242/dev.128.8.1253

[39] Soriano P (1999) Generalized lacZ expression with the ROSA26 Cre reporter strain. Nat Genet 21: 70–71. 9916792 10.1038/5007

[40] Lewandoski M, Wassarman KM, Martin GR (1997) Zp3-cre, a transgenic mouse line for the activation or inactivation of loxP-flanked target genes specifically in the female germ line. Curr Biol 7: 148–151. 9016703 10.1016/s0960-9822(06)00059-5

[41] Schaeren-Wiemers N, Gerfin-Moser A (1993) A single protocol to detect transcripts of various types and expression levels in neural tissue and cultured cells: in situ hybridization using digoxigenin-labelled cRNA probes. Histochemistry 100: 431–440. 7512949 10.1007/BF00267823

[42] Harlow E, Lane D (1999) Using Antibodies: A Laboratory Manual. Plainview, NY: Cold Spring Harbor Lab. Press.

[43] Matsunami H, Takeichi M (1995) Fetal brain subdivisions defined by R- and E-cadherin expressions: evidence for the role of cadherin activity in region-specific, cell-cell adhesion. Dev Biol 172: 466–478. 8612964 10.1006/dbio.1995.8029

[44] Lustig B, Jerchow B, Sachs M, Weiler S, Pietsch T, et al (2002) Negative feedback loop of Wnt signaling through upregulation of conductin/axin2 in colorectal and liver tumors. Mol Cell Biol 22: 1184–1193. 11809809 10.1128/MCB.22.4.1184-1193.2002 PMC134640

[45] Jho E-H, Zhang T, Domon C, Joo CK, Freund J-N, et al (2002) Wnt/β-catenin/Tcf signaling induces the transcription of Axin2, a negative regulator of the signaling pathway. Mol Cell Biol 22: 1172–1183. 11809808 10.1128/MCB.22.4.1172-1183.2002 PMC134648

[46] Burns CJ, Zhang J, Brown EC, Van Bibber AM, Van Es J, et al (2008) Investigation of Frizzled-5 during embryonic neural development in mouse. Dev Dyn 237: 1614–1626. 18489003 10.1002/dvdy.21565 PMC2562763

[47] Fuhrmann S, Riesenberg AN, Mathiesen AM, Brown EC, Vetter ML, et al (2009) Characterization of a transient TCF/LEF-responsive progenitor population in the embryonic mouse retina. Invest Ophthalmol Vis Sci 50: 432–440. 18599572 10.1167/iovs.08-2270 PMC2615067

[48] Nornes HO, Dressler GR, Knapik EW, Deutsch U, Gruss P (1990) Spatially and temporally restricted expression of Pax2 during murine neurogenesis. Development 109: 797–809. 1977575 10.1242/dev.109.4.797

[49] Torres M, Gomez-Pardo E, Gruss P (1996) Pax2 contributes to inner ear patterning and optic nerve trajectory. Development 122: 3381–3391. 8951055 10.1242/dev.122.11.3381

[50] Eiraku M, Adachi T, Sasai Y (2012) Relaxation-expansion model for self-driven retinal morphogenesis: a hypothesis from the perspective of biosystems dynamics at the multi-cellular level. Bioessays 34: 17–25. 22052700 10.1002/bies.201100070 PMC3266490

[51] Amano M, Ito M, Kimura K, Fukata Y, Chihara K, et al (1996) Phosphorylation and activation of myosin by Rho-associated kinase (Rho-kinase). J Biol Chem 271: 20246–20249. 8702756 10.1074/jbc.271.34.20246

[52] Cho S-H, Cepko CL (2006) Wnt2b/β-catenin-mediated canonical Wnt signaling determines the peripheral fates of the chick eye. Development 133: 3167–3177. 16854977 10.1242/dev.02474

[53] Liu H, Thurig S, Mohamed O, Dufort D, Wallace VA (2006) Mapping canonical Wnt signaling in the developing and adult retina. Invest Ophthalmol Vis Sci 47: 5088–5097. 17065530 10.1167/iovs.06-0403

[54] Tang K, Xie X, Park JI, Jamrich M, Tsai S, et al (2010) COUP-TFs regulate eye development by controlling factors essential for optic vesicle morphogenesis. Development 137: 725–734. 20147377 10.1242/dev.040568 PMC2827684

[55] Xiang M, Zhou L, Macke JP, Yoshioka T, Hendry SH, et al (1995) The Brn-3 family of POU-domain factors: primary structure, binding specificity, and expression in subsets of retinal ganglion cells and somatosensory neurons. J Neurosci 15: 4762–4785. 7623109 10.1523/JNEUROSCI.15-07-04762.1995 PMC6577904

[56] Mears AJ, Kondo M, Swain PK, Takada Y, Bush RA, et al (2001) Nrl is required for rod photoreceptor development. Nat Genet 29: 447–452. 11694879 10.1038/ng774

[57] Furukawa T, Morrow EM, Cepko CL (1997) Crx, a novel otx-like homeobox gene, shows photoreceptor-specific expression and regulates photoreceptor differentiation. Cell 91: 531–541. 9390562 10.1016/s0092-8674(00)80439-0

[58] Ng L, Hurley JB, Dierks B, Srinivas M, Salto C, et al (2001) A thyroid hormone receptor that is required for the development of green cone photoreceptors. Nat Genet 27: 94–98. 11138006 10.1038/83829

[59] Dyer MA, Livesey FJ, Cepko CL, Oliver G (2003) Prox1 function controls progenitor cell proliferation and horizontal cell genesis in the mammalian retina. Nat Genet 34: 53–58. 12692551 10.1038/ng1144

[60] Fujitani Y, Fujitani S, Luo H, Qiu F, Burlison J, et al (2006) Ptf1a determines horizontal and amacrine cell fates during mouse retinal development. Development 133: 4439–4450. 17075007 10.1242/dev.02598

[61] Nakhai H, Sel S, Favor J, Mendoza-Torres L, Paulsen F, et al (2007) Ptf1a is essential for the differentiation of GABAergic and glycinergic amacrine cells and horizontal cells in the mouse retina. Development 134: 1151–1160. 17301087 10.1242/dev.02781

[62] Dorsky RI, Sheldahl LC, Moon RT (2002) A transgenic Lef1/β-catenin-dependent reporter is expressed in spatially restricted domains throughout zebrafish development. Dev Biol 241: 229–237. 11784107 10.1006/dbio.2001.0515

[63] Van Raay TJ, Moore KB, Iordanova I, Steele M, Jamrich M, et al (2005) Frizzled 5 signaling governs the neural potential of progenitors in the developing Xenopus retina. Neuron 46: 23–36. 15820691 10.1016/j.neuron.2005.02.023

[64] Bumsted KM, Barnstable CJ (2000) Dorsal retinal pigment epithelium differentiates as neural retina in the microphthalmia (mi/mi) mouse. Invest Ophthalmol Vis Sci 41: 903–908. 10711712

[65] Schepsky A, Bruser K, Gunnarsson GJ, Goodall J, Hallsson JH, et al (2006) The microphthalmia-associated transcription factor Mitf interacts with beta-catenin to determine target gene expression. Mol Cell Biol 26: 8914–8927. 17000761 10.1128/MCB.02299-05 PMC1636837

[66] Yasumoto K, Takeda K, Saito H, Watanabe K, Takahashi K, et al (2002) Microphthalmia-associated transcription factor interacts with LEF-1, a mediator of Wnt signaling. EMBO J 21: 2703–2714. 12032083 10.1093/emboj/21.11.2703 PMC126018

[67] Raymond SM, Jackson IJ (1995) The retinal pigmented epithelium is required for development and maintenance of the mouse neural retina. Curr Biol 5: 1286–1295. 8574586 10.1016/s0960-9822(95)00255-7

[68] Ouyang M, Lu S, Kim T, Chen CE, Seong J, et al (2013) N-cadherin regulates spatially polarized signals through distinct p120ctn and β-catenin-dependent signalling pathways. Nat Commun 4: 1589. 23481397 10.1038/ncomms2560 PMC3602931

[69] Zakin L, Mazan S, Maury M, Martin N, Guenet J-L, et al (1998) Structure and expression of Wnt13, a novel mouse Wnt2 related gene. Mech Develop 73: 107–116. 10.1016/s0925-4773(98)00040-9 9545553

[70] Tsukiyama T, Yamaguchi TP (2012) Mice lacking Wnt2b are viable and display a postnatal olfactory bulb phenotype. Neurosci Lett 512: 48–52. 22326927 10.1016/j.neulet.2012.01.062 PMC3298629

[71] Zhou CJ, Wang YZ, Yamagami T, Zhao T, Song L, et al (2010) Generation of Lrp6 conditional gene-targeting mouse line for modeling and dissecting multiple birth defects/congenital anomalies. Dev Dyn 239: 318–326. 19653321 10.1002/dvdy.22054

[72] Mao J, Wang J, Liu B, Pan W, Farr GH 3rd, et al (2001) Low-density lipoprotein receptor-related protein-5 binds to Axin and regulates the canonical Wnt signaling pathway. Mol Cell 7: 801–809. 11336703 10.1016/s1097-2765(01)00224-6

[73] Pinson K, Brennan J, Monkley S, Avery B, Skarnes W (2000) An LDL-receptor-related protein mediates Wnt signalling in mice. Nature 407: 535–538. 11029008 10.1038/35035124

[74] Tamai K, Semenov M, Kato Y, Spokony R, Liu C, et al (2000) LDL-receptor-related proteins in Wnt signal transduction. Nature 407: 530–535. 11029007 10.1038/35035117

[75] Liu C, Bakeri H, Li T, Swaroop A (2012) Regulation of retinal progenitor expansion by Frizzled receptors: implications for microphthalmia and retinal coloboma. Hum Mol Genet 21: 1848–1860. 22228100 10.1093/hmg/ddr616 PMC3313798

[76] Van Raay TJ, Vetter ML (2004) Wnt/frizzled signaling during vertebrate retinal development. Dev Neurosci 26: 352–358. 15855764 10.1159/000082277

