# Supplementary figures and images for: Canonical Wnt/β-Catenin Signalling Is Essential for Optic Cup Formation
Source: PLoS One. 2013 Dec 4;8(12):e81158. doi: 10.1371/journal.pone.0081158 (PMC3852023; doi:10.1371/journal.pone.0081158)

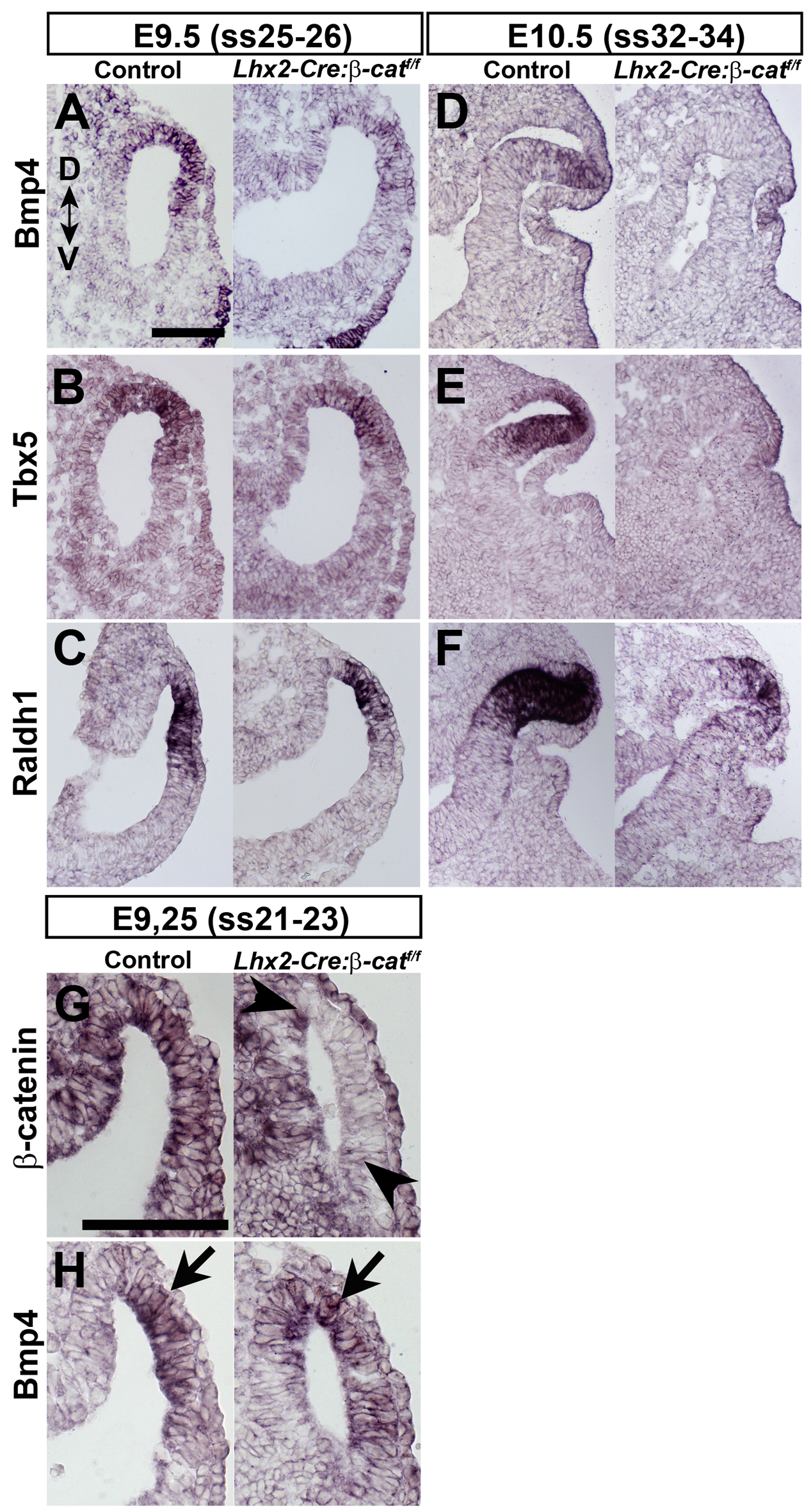

Supplement: Figure S1 — Maintenance but not induction of dorsal identity of the optic vesicle is dependent on β-catenin. (A–C) In situ hybridization analyses on coronal sections of E9.5 (ss25–26) optic vesicles from control (left panels) and Lhx2-Cre:β-cateninflox/flox embryos (right panels). (D–F) In situ hybridization analyses on coronal sections of E10.5 (ss32–34) optic cups from control (left panels) and Lhx2-Cre:β-cateninflox/flox embryos (right panels). (G, H) In situ hybridization analyses on coronal sections of E9.25 (ss21–23) optic vesicles from control embryos (left panels) and Lhx2-Cre:β-cateninflox/flox embryos (right panels). Arrow heads indicate the boundaries where β-catenin has been inactivated in the optic vesicle. Arrows indicate Bmp4 expression. Dorsal-ventral (D–V) orientation for all panels is indicated in A. Scale bars: (A–F and G, H) 100 µm. (TIF) [file pone.0081158.s001.tif]

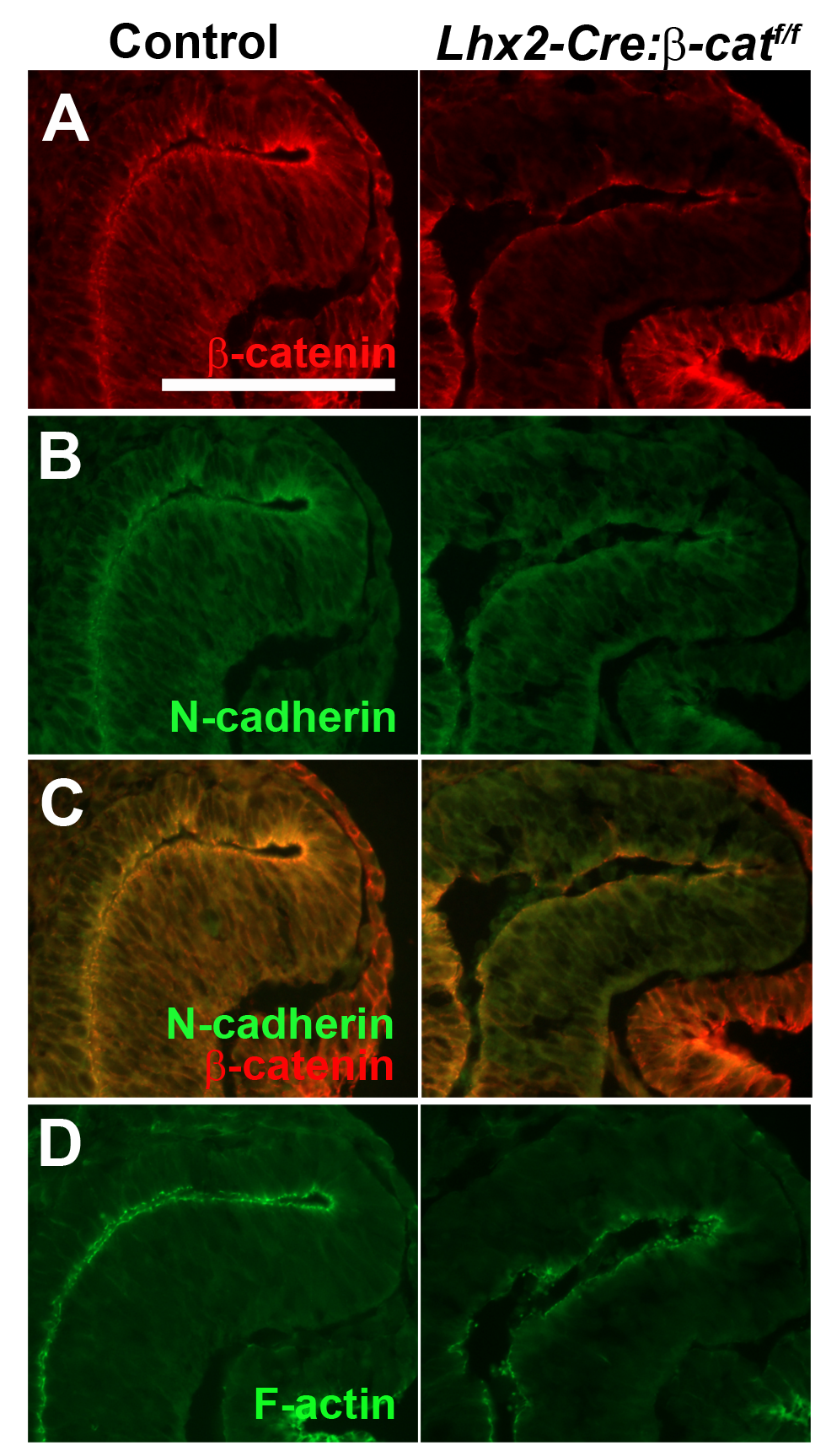

Supplement: Figure S2 — β-catenin protein remains associated with N-cadherin and F-actin in the mutant optic vesicle at E10.5. (A–D) Immunohistochemical analyses for cellular localisation of the indicated proteins on coronal sections of E10.5 (ss 33–35) optic vesicles from control embryos (left panels) and Lhx2-Cre:β-cateninflox/flox embryos (right panels). (A) and (B) are merged in (C) to show co-localisation (yellow) on the apical side of the cells in the optic vesicle. (D) Serial section following those in (A–C). Note that while the RPE and the neural retina have been specified in the control embryo (left panels), the corresponding structure in the mutant embryo contains almost exclusively cells of neural retinal fate at this developmental stage (see Figure 3L, 3M, and 3N). Scale bar: 100 µm. (TIF) [file pone.0081158.s002.tif]

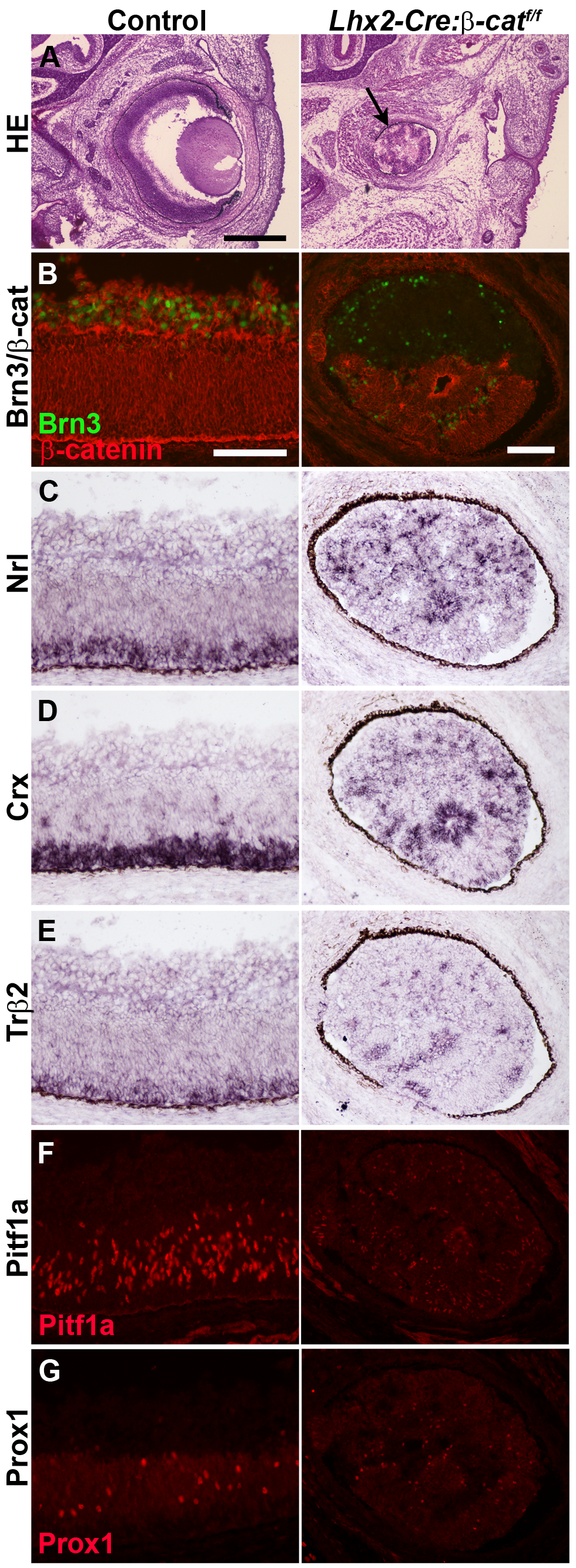

Supplement: Figure S3 — Retinal cell classes can develop independent of β-catenin. (A) Hematoxylin/eosin staining of coronal sections of an E18.5 control embryo (left panel) and an Lhx2-Cre:β-cateninflox/flox mutant embryo (right panel). Arrow indicates the optic rudiment that can develop in mutant embryos (right panel). (B, F, G) Immunohistochemical analyses for the presence of the indicated proteins on coronal sections of control (left panels) and mutant embryos (right panels). (C–E) In situ hybridization analyses for gene expression analysis of the indicated genes on coronal sections of control (left panels) and mutant embryos (right panels). All the sections from the mutant embryo have been analysed for β-catenin expression on a consecutive section to ensure that the distribution of β-catenin+ and β-catenin− cells shown in panel (B), is maintained in all panels. Scale bars: (A) 500 µm. (B–G, left and right panels) 100 µm. (TIF) [file pone.0081158.s003.tif]
